# Association analysis using somatic mutations

**DOI:** 10.1371/journal.pgen.1007746

**Published:** 2018-11-02

**Authors:** Yang Liu, Qianchan He, Wei Sun

**Affiliations:** 1 Department of Mathematics and Statistics, Wright State University, Dayton, Ohio, United States of America; 2 Biostatistics Program, Public Health Sciences Division, Fred Hutchinson Cancer Research Center, Seattle, Washington, United States of America; Case Western Reserve University, UNITED STATES

## Abstract

Somatic mutations drive the growth of tumor cells and are pivotal biomarkers for many cancer treatments. Genetic association analysis using somatic mutations is an effective approach to study the functional impact of somatic mutations. However, standard regression methods are not appropriate for somatic mutation association studies because somatic mutation calls often have non-ignorable false positive rate and/or false negative rate. While large scale association analysis using somatic mutations becomes feasible recently—thanks for the improvement of sequencing techniques and the reduction of sequencing cost—there is an urgent need for a new statistical method designed for somatic mutation association analysis. We propose such a method with computationally efficient software implementation: Somatic mutation Association test with Measurement Errors (SAME). SAME accounts for somatic mutation calling uncertainty using a likelihood based approach. It can be used to assess the associations between continuous/dichotomous outcomes and individual mutations or gene-level mutations. Through simulation studies across a wide range of realistic scenarios, we show that SAME can significantly improve statistical power than the naive generalized linear model that ignores mutation calling uncertainty. Finally, using the data collected from The Cancer Genome Atlas (TCGA) project, we apply SAME to study the associations between somatic mutations and gene expression in 12 cancer types, as well as the associations between somatic mutations and colon cancer subtype defined by DNA methylation data. SAME recovered some interesting findings that were missed by the generalized linear model. In addition, we demonstrated that mutation-level and gene-level analyses are often more appropriate for oncogene and tumor-suppressor gene, respectively.

## Author summary

Cancer is a genetic disease that is driven by the accumulation of somatic mutations. Association studies using somatic mutations is a powerful approach to identify the potential impact of somatic mutations on molecular or clinical features. One challenge for such tasks is the non-ignorable somatic mutation calling errors. We have developed a statistical method to address this challenge and applied our method to study the gene expression traits associated with somatic mutations in 12 cancer types. Our results show that some somatic mutations affect gene expression in several cancer types. In particular, we show that the associations between gene expression traits and TP53 gene level mutation reveal some similarities across a few cancer types.

## Introduction

Somatic mutations play a central role in the development and progression of cancer. Associations between somatic mutations and molecular/clinical outcomes can provide important insights into cancer etiology or the mechanism of tumor growth, and potentially contribute to precision cancer therapy. Despite the functional importance of somatic mutations, few computational methods have been developed for association studies using somatic mutations. There are probably two reasons for this. First, since somatic mutation data are relatively new, most efforts were spent on bioinformatic challenges such as somatic mutation calling and functional annotations, e.g., inference of driver mutations [1–3], or estimation of cancer subtypes using somatic mutations [4, 5]. Second, systematic studies of somatic mutations in large observational studies are not feasible until recently, thanks for the drop of sequencing cost and the improved capability to handle formalin-fixed paraffin-embedded (FFPE) tissue samples. While these challenges on sequencing tumor samples and calling mutations have been addressed, a limiting factor to harvest the rich information of somatic mutation associations is appropriate statistical methods for data analysis.

A unique feature of somatic mutations, in contrast to germline mutations, is the difficulty to confidently call mutations from sequencing data. A major factor that contributes to this challenge is that a tumor sample is often a mixture of tumor cells and non-tumor cells (e.g., infiltrating immune cells) and a somatic mutation may only occur in a subset of the tumor cells, known as intra-tumor heterogeneity [6]. Therefore the signals of a somatic mutation may be visible only in a small proportion of sequence reads, and it is challenging to separate such weak signals from sequencing errors or DNA damages caused by FFPE [7]. Another factor that limits mutation call availability/accuracy is low coverage of sequencing reads, particularly in whole genome sequencing data. Although many methods have been developed for somatic mutation calling [8–11], there is no consensus on the best variant calling algorithm. The general recommendation is to take the intersection of the mutations called by a few methods, followed by additional filters [12, 13]. Such a strategy reduces false positive rate, but at the price of inflated false negative rate. Therefore it is important to account for somatic mutation calling uncertainty in association studies.

Such uncertainty of somatic mutation calling renders association methods for germline genetic variants inappropriate for somatic mutation associations. Generalized linear models are the most commonly used tools to assess germline genetic associations, for example, linear model for continuous traits and logistic regression for binary traits. Such methods do not account for mutation calling uncertainty. A few germline genetic association methods have been developed when the germline genomic features have inherent uncertainty, for example, for haplotype association [14, 15] or for case-control associations with systematic difference between cases and controls [16]. However, these methods are designed for specific problems and are not applicable to somatic mutation association studies.

A few earlier works have studied the associations between somatic mutations and gene expression using gene-level mutation [17], by integrating gene-gene interaction networks [18], or by a meta-analysis across multiple cancer types [19]. However, none of these works has considered the uncertainty of somatic mutation calling. In this paper, we propose a Somatic mutation Association test with Measurement Errors (SAME), which accounts for somatic mutation calling uncertainty by modeling the true somatic mutation status as a latent variable and exploiting read count data to augment the mutation calls. We develop two versions of this test, one for mutation-level analysis using a single somatic mutation (mSAME) and the other one for gene-level analysis using multiple mutations within a gene (gSAME). We have implemented SAME in an R package, and it is computationally efficient enough for genome-wide analysis. We evaluated the performance of SAME through extensive simulations and a real data application using the data from 12 cancer types of The Cancer Genome Atlas (TCGA) project. Our results demonstrated that SAME controls type I error and has improved statistical power compared to the competing methods that ignore somatic mutation calling uncertainty.

## Materials and methods

### mSAME model

We first describe the mSAME test that works on a single somatic mutation. To simplify notations, we omit the index for somatic mutations in the following discussions. For a specific somatic mutation, we denote the mutation call and true mutation status in the *i*-th sample by *O*_*i*_ and *S*_*i*_, respectively, where 1 ≤ *i* ≤ *n* and *n* is sample size. *S*_*i*_ equals to 1 if this mutation is present in the *i*-th sample, and 0 otherwise. The value of *O*_*i*_ depends on the read-depth information. Let the read-depth and the number of alternative reads of this mutation in the *i*-th sample be *D*_*i*_ and *A*_*i*_, respectively. A somatic mutation can be called only if there is enough coverage, i.e, *O*_*i*_ = 0 or 1 as mutation call indicator if *D*_*i*_ ≥ *d*_0_, and *O*_*i*_ is unobserved if *D*_*i*_ < *d*_0_, where *d*_0_ is a threshold used in the mutation calling process. Denote the outcome variable of the *i*-th sample by *Y*_*i*_ and the set of additional covariates by *x*_*i*_. Let *ρ*_0_ = *P*(*S*_*i*_ = 0) and *ρ*_1_ = 1 − *ρ*_0_ = *P*(*S*_*i*_ = 1), then the likelihood for the observed data can be written as

$$\begin{array}{c} \begin{array}{cc} L & =\prod_{i=1}^{n}\sum_{j=0}^{1}\rho_{j}f_{Y,A,D,O}\left(Y_{i},A_{i},D_{i},O_{i}|S_{i}=j\right)\\ & =\prod_{i=1}^{n}\sum_{j=0}^{1}\rho_{j}f_{Y}\left(Y_{i}|S_{i}=j\right)f_{A,D,O}\left(A_{i},D_{i},O_{i}|S_{i}=j\right), \end{array} \end{array}$$ (1)

where *f*_*T*_ denotes the density function for random variable *T*.

We further assume that the conditional distribution of *Y*_*i*_ given *S*_*i*_ (i.e., *f*_*Y*_(*Y*_*i*_|*S*_*i*_ = *j*) in Eq (1)) can be modeled by a generalized linear model with mean

$$\begin{array}{c} E\left(Y_{i}\right)=g^{-1}\left(x_{i}^{T}\alpha +S_{i}\beta \right), \end{array}$$ (2)

and a dispersion parameter *ϕ*, where *g*(⋅) is a link function, and *α*, *β* are the regression coefficients. We are interested in the association testing problem *H*_0_: *β* = 0. For continuous outcomes, we can write *f*_*Y*_(*Y*_*i*_|*S*_*i*_) as a normal density with the identity link function *g*(*t*) = *t*. For binary outcomes, we write *f*_*Y*_(*Y*_*i*_|*S*_*i*_) as a Bernoulli density using the logit link function *g*(*t*) = log(*t*/(1 − *t*)).

For the distribution of read counts and observed mutation calls (i.e., *f*_*A*,*D*,*O*_(*A*_*i*_, *D*_*i*_, *O*_*i*_|*S*_*i*_ = *j*) in Eq (1)), we use beta-binomial distributions to model allele-specific read counts *A*_*i*_ given *D*_*i*_, *O*_*i*_ and *S*_*i*_, and use a Bernoulli distribution to model *O*_*i*_ given *S*_*i*_. Beta-binomial distributions have been used to model allele-specific read counts from ChIP-seq [20], RNA-seq [21], DNA sequencing [22], and somatic mutations [23, 24]. The Bernoulli likelihood of observed somatic mutation calls given true somatic mutation status has been used to model somatic mutation calls from single cell DNA sequencing data [25, 26]. These previous work have shown that these distributions are appropriate for real data. We have also compared the distributions of observed read counts versus expected ones from beta-binomial model fit and they agree very well (Fig S1 in S1 Appendix).

We denote the unknown parameters in the model by *θ* and the likelihood-ratio test statistic for the mSAME model is

$$\begin{array}{c} T=-2[\log L\left({\hat{\theta }}_{0};Y,A,D,O\right)-\log L\left(\hat{\theta };Y,A,D,O\right)], \end{array}$$ (3)

where $\hat{\theta }$ is the maximum likelihood estimator of *θ* in the whole parameter space, and ${\hat{\theta }}_{0}$ is the maximum likelihood estimator of *θ* under *H*_0_: *β* = 0. All the technical details for the likelihood function and parameter estimation can be found in Section 1.1-1.4 of S1 Appendix. Under *H*_0_, the test statistic *T* asymptotically follows a Chi-square distribution with degree of freedom 1, thus we can reject *H*_0_ if $T>\chi_{1}^{2}\left(1-\xi \right)$ where $\chi_{1}^{2}\left(1-\xi \right)$ is the (1 − *ξ*) quantile of this Chi-square distribution.

### gSAME model

Next we discuss our gSAME model that aggregates the information of multiple somatic mutation loci within a gene (or any arbitrarily defined unit) for association testing. We start by defining some notations. Suppose that there are *p* mutation loci within a gene of interest, and we drop the index for gene for notational convenience. We use superscripts ^*m*^ and ^*g*^ to denote mutation-level and gene-level data, respectively. We denote the observed mutation calls for the *i*-th sample by $O_{i}^{m}=\left\{O_{i1}^{m},\cdots ,O_{ip}^{m}\right\}$, the read-depth and the number of the alternative reads by $D_{i}^{m}=\left\{D_{i1}^{m},\cdots ,D_{ip}^{m}\right\}$ and $A_{i}^{m}=\left\{A_{i1}^{m},\cdots ,A_{ip}^{m}\right\}$, respectively. Analogously, we denote the underlying true mutation status by $S_{i}^{m}=\left\{S_{i1}^{m},\cdots ,S_{ip}^{m}\right\}$. We define the gene-level mutation status to be 1 if there is one or more mutations within this gene:

$$\begin{array}{c} S_{i}^{g}=\{\begin{array}{cc} 1 & \text{if}\;\text{any}\;S_{ij}^{m}=1,\\ 0 & \text{if}\;\text{all}\;S_{ij}^{m}=0. \end{array} \end{array}$$ (4)

The outcome variable *Y*_*i*_ and the covariates *x*_*i*_ are defined as before. In gene-level analysis, we model *Y*_*i*_ as a function of $S_{i}^{g}$ and *x*_*i*_. Then the likelihood function is

$$\begin{array}{c} \begin{array}{cc} L & =\prod_{i=1}^{n}\sum_{j=0}^{1}\rho_{j}^{g}f_{Y,A,D,O}\left(Y_{i},A_{i}^{m},D_{i}^{m},O_{i}^{m}|S_{i}^{g}=j\right)\\ & =\prod_{i=1}^{n}\sum_{j=0}^{1}\rho_{j}^{g}f_{Y}\left(Y_{i}|S_{i}^{g}=j\right)f_{A,D,O}\left(A_{i}^{m},D_{i}^{m},O_{i}^{m}|S_{i}^{g}=j\right), \end{array} \end{array}$$ (5)

where $\rho_{0}^{g}=P\left(S_{i}^{g}=0\right)$ and $\rho_{1}^{g}=1-\rho_{0}^{g}=P\left(S_{i}^{g}=1\right)$.

Since read count data (i.e., $D_{i}^{m}$ and $A_{i}^{m}$) and mutation calls ($O_{i}^{m}$) are collected for each mutated locus, their distributions are modeled given $S_{i}^{m}$. Then the remaining steps to complete this likelihood is to model $S_{i}^{m}$ conditional on $S_{i}^{g}$. When $S_{i}^{g}=0$, it is clear that $S_{ij}^{m}=0$ for all the *p* mutations. When $S_{i}^{g}=1$, $S_{i}^{m}$ can have 2^*p*^ − 1 possible values, which is computationally onerous to enumerate for large *p*. We notice that in practice, it is impossible to call a somatic mutation if the corresponding number of alternative reads equals to 0. Hence to reduce computational burden, we assume that the *j*-th mutation may occur only if $A_{ij}^{m}>0$, otherwise we assign $S_{ij}^{m}=0$ directly. Thus the number of the combinations is limited to $2^{m_{i}}-1$, where *m*_*i*_ is the number of mutations with $A_{ij}^{m}>0$.

Let *θ*^*g*^ be the unknown parameters in the gSAME model. The likelihood ratio test statistic of gSAME model for testing the effect of somatic mutation $S_{i}^{g}$ is

$$\begin{array}{c} T=-2[\log L\left({\hat{\theta }}_{0}^{g};Y,A^{m},D^{m},O^{m}\right)-\log L\left({\hat{\theta }}^{g};Y,A^{m},D^{m},O^{m}\right)], \end{array}$$ (6)

where ${\hat{\theta }}^{g}$ is the estimator of *θ*^*g*^ in the whole parameter space, and ${\hat{\theta }}_{0}^{g}$ is the estimator of *θ*^*g*^ under *H*_0_. All the technical details for the likelihood function and parameter estimation can be found in Section 1.5-1.6 of S1 Appendix.

## Results

### Simulation studies

#### Simulations for mSAME model

We generated a dataset of *n* = 400 samples. For the *i*-th sample, we simulated the true somatic mutation value *S*_*i*_ by a Bernoulli distribution with success probability *ρ*_1_, and we vary *ρ*_1_ in different simulation setups. A continuous outcome *Y*_*i*_ was simulated by *Y*_*i*_ = 1 + *x*_*i*_ + *βS*_*i*_ + *ϵ*_*i*_, where *x*_*i*_ and *ϵ*_*i*_ were generated by the standard normal distribution independently. A dichotomous outcome *Y*_*i*_ was simulated from a Bernoulli distribution with success probability *p*_*i*_, and log(*p*_*i*_/(1 − *p*_*i*_)) = −0.5 + *x*_*i*_ + *βS*_*i*_. Based on the true mutation value *S*_*i*_, we simulated the observed mutation *O*_*i*_ by the Bernoulli distributions specified in equation (3) of S1 Appendix, with the sensitivity and specificity being *γ*_1_ = 0.9 and *γ*_0_ = 0.98, respectively. These choices of sensitivity and specificity are based on the evaluation of somatic mutation callers in a previous study [12]. It is desirable to generate *O*_*i*_ by actually performing somatic mutation calling. However, we did not pursue on this direction because it would require generation of bam files and simulating many factors, such as sequencing quality scores, mapping quality scores, clustering of reads due to amplification artifacts, and strand bias, and we are not aware of any existing tool to generate such bam files.

We simulated read-depth of somatic mutations to mimic the read-depth data observed in a TCGA exome-seq dataset (across 133,463 somatic mutations in 433 colon cancer patients). More details of this dataset are presented in the following sections on TCGA data analysis and S3 Appendix. In exome-seq data, read-depth varies across genomic loci and across samples, and thus we simulated read-depth in two steps. First, we simulated mean read-depth for each mutation by a negative binomial distribution with mean *μ* = 113 and over-dispersion *ϕ* = 3.28, so the standard deviation is $\sqrt{\mu +\mu^{2}/\phi }≃63.3$. Next, for each mutation, we simulated read-depth across samples by a negative binomial distribution with mean value simulated in the first step, and over-dispersion 1.9. When *D*_*i*_ ≥ *d*_0_ = 20, we simulated *A*_*i*_ by a beta-binomial distribution specified in equation (2) of S1 Appendix, with parameters (*π*_00_, *π*_01_, *π*_10_, *π*_11_) = (0.001, 0.002, 0.1179, 0.3207) and (*φ*_00_, *φ*_01_, *φ*_10_, *φ*_11_) = (0.0006, 0.3457, 0.0001, 0.1018). Later in simulation studies, we estimate these parameters based on 50 simulated somatic mutations across 400 samples, and the estimates are fairly accurate. When *D*_*i*_ < 20, the number of alternative reads *A*_*i*_ was generated by a beta-binomial distribution (see equation (4) of S1 Appendix) with parameters *π*_0_ = 0.001, *φ*_0_ = 0.001, *π*_1_ = 0.146, and *φ*_1_ = 0.10. The parameters of these negative binomial and beta binomial distributions are all estimated from the TCGA colon cancer dataset.

Using this simulated dataset, we compare the performance of mSAME and a naive generalized linear model, in terms of type I error and power for testing the hypothesis *β* = 0. The generalized linear model does not account for somatic mutation calling uncertainty, but simply treats the observed somatic mutation call *O*_*i*_ as the true somatic mutation status and performs a Wald test on the regression coefficient *β*.

**Fig 1:**
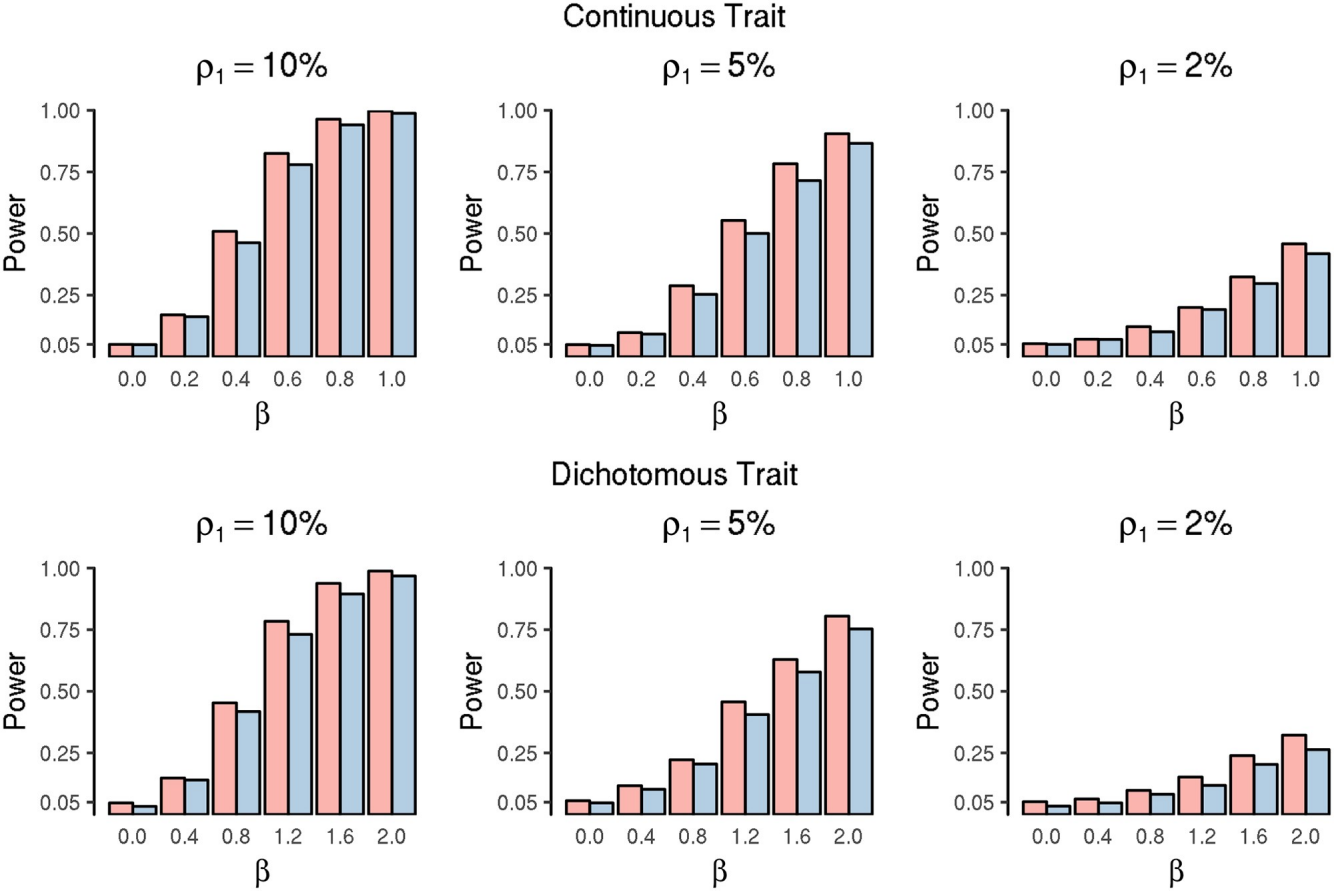
Power comparison of mSAME (red bars) and GLM (blue bars) for mutation-level simulations.

Across different simulation settings, we considered various mutation frequencies *ρ*_1_ = 0.02, 0.05, 0.10 and effect size *β*, and evaluate the performance over 1,000 replicates. We set *β* = 0 to evaluate the type I error at the significance level 0.05. For the power performance, we set *β* = 0.2, 0.4, 0.6, 0.8, 1.0 for the continuous trait and *β* = 0.4, 0.8, 1.2, 1.6, 2.0 for the binary trait. In all the scenarios, the type I errors of both methods are well controlled, and the mSAME has higher power than GLM for all simulation settings and for both continuous and binary traits (Fig 1, Table S1 in S2 Appendix). In addition, mSAME has more accurate estimates of *β*, evaluated by the mean square error (MSE) (Fig S2 in S2 Appendix).

#### Simulations for gSAME model

For the gene-level mutation analysis, we are interested in the association between an outcome *Y* and a gene-level mutation $S_{i}^{g}$. We assume that there are *p* = 10 mutations within the gene, and denote the frequency of the gene-level mutation as $P\left(S_{i}^{g}=1\right)=\rho_{1}^{g}$. We set the sample size *n* = 400. For the *i*-th sample, we generated the true mutation values $S_{ij}^{m},j=1,\cdots ,p$ independently by a Bernoulli distribution with $P\left(S_{ij}^{m}=1\right)=1-{\left(1-\rho_{1}^{g}\right)}^{1/p}$. Then the gene-level mutation value $S_{i}^{g}$ can be obtained by collapsing mutation level data (Eq (4)). The continuous outcome variable *Y*_*i*_ was simulated by $Y_{i}=1+x_{i}+\beta S_{i}^{g}+{∊}_{i}$, where *x*_*i*_ and *ϵ*_*i*_ were simulated by the standard normal distribution independently. The binary outcome *Y*_*i*_ was simulated from a Bernoulli distribution so that $\text{logit}\left[p\left(Y_{i}=1\right)\right]=-0.5+x_{i}+\beta S_{i}^{g}$. In addition, for the *j*-th mutation, the observed mutation call $O_{ij}^{m}$, the read-depth $D_{ij}^{m}$ and the number of alternative reads $A_{ij}^{m}$ were simulated based on the true mutation value $S_{ij}^{m}$, following the same procedure as the mutation-level simulations. When simulating *O*_*ij*_ within a gene, we randomly chose the specificity to be 0.98 or 1 with equal probabilities and randomly chose the sensitivity to be 0.9 or 1 with equal probabilities.

We compared the performance of gSAME with a naive GLM method. For the naive GLM method, we regress the response *Y*_*i*_ on *x*_*i*_ and the observed gene-level somatic mutation $O_{i}^{g}$, where $O_{i}^{g}$ is defined as

$$\begin{array}{c} O_{i}^{g}=\{\begin{array}{cc} 1 & \text{if}\;\text{any}\;O_{ij}^{m}=1,\\ 0 & \text{if}\;\text{all}\;O_{ij}^{m}=0. \end{array} \end{array}$$ (7)

**Fig 2:**
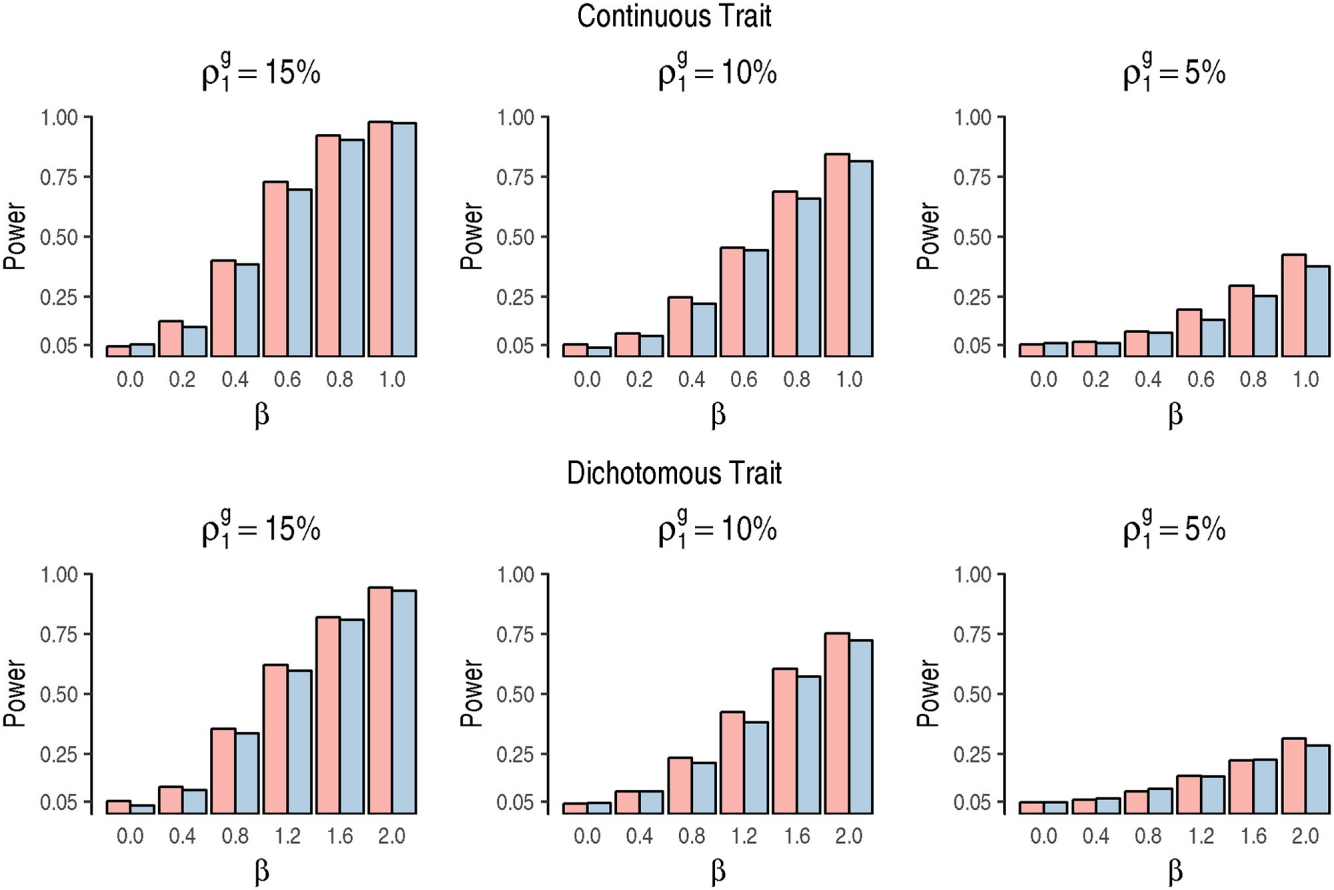
Power comparison of gSAME (red bars) and GLM (blue bars) for gene-level simulations.

For mutation-level associations, we have considered the mutation frequencies of *ρ*_1_ = 0.02, 0.05, or 0.10. Since the gene-level mutation frequencies are usually higher than mutation level mutation frequencies, for gene-level mutation, we set $\rho_{1}^{g}=0.05$, 0.10, or 0.15. The regression coefficient *β* was set to be the same as in the mutation-level analysis. All the results were evaluated over 1,000 replicates. Overall, both gSAME and GLM control the Type I error, and gSAME always has higher power than GLM (Fig 2, Table S2 in S2 Appendix), and more accurate estimates of *β* (Fig S2 in S2 Appendix). Given the same mutation frequency, the power of gene-level analysis is lower than mutation-level analysis because the mutation-level measurement errors aggregate and become larger at gene-level.

In conclusion, SAME has higher power than the naive GLM approach that ignores mutation calling uncertainty, even if the mutation calling is relatively accurate (sensitivity 0.9 and specificity 0.98) and read-depth is relatively high (average read-depth of 113). This is because our model accounts for the imperfect sensitivity and specificity, and read-depth can be low for some genes in some samples, due to uneven coverage of exome-seq data (Fig S3 in S2 Appendix). When the mutation calling becomes less accurate (e.g., sensitivity 0.9 and specificity 0.95) or the read-depth becomes lower (e.g., for whole genome sequencing data, where the typical read-depth is 20x to 40x), SAME has even larger power gain than GLM. For example, in an additional simulation setup, we simulated read-depth by a negative binomial distribution with mean 40 and over-dispersion 1.9, resembling a whole-genome sequencing situation. For a continuous trait, with mutation frequency 5% and effect size *β* = 0.2 or 0.4, the power gain of mSAME vs. GLM is around 40% for this whole-genome sequencing simulation setup (Fig S4 in S2 Appendix). In contrast, our main simulation setup resembles an exome-seq data where the power gain is around 10%. See S2 Appendix for more details of additional simulation setup and results.

### TCGA colon cancer eQTL analysis

We applied the proposed mSAME and gSAME methods as well as GLM to study the associations between somatic mutations and genome-wide gene expression in TCGA colon cancer patients. Briefly, we downloaded the bam files of exome-seq data for paired tumor-normal samples from NCI Genomic Data Commons (GDC) Data Portal. We called somatic mutations using the intersection of MuTect and Strelka, followed by the read-depth filter to keep those mutations with read-depth ≥20 in both tumor and paired-normal samples (Section 3.1 in S3 Appendix). Colon cancer patients can be separated into two subtypes based on mutation load [27]. We classified a sample as hyper-mutated if it has more than 375 non-silent mutations and this cutoff is chosen to separate the two modes of the distribution of mutation load (Fig S9 in S3 Appendix). Our analysis requires allele-specific read counts for each mutation across all samples. While collecting such information, we noticed that 24 samples have much smaller number of allele-specific read counts than the remaining samples and we removed them from our data analysis (Section 3.3 in S3 Appendix).

For gene expression data, we downloaded the .htseq.counts files from NCI GDC, which include the number of RNA-seq reads mapped to 60,483 genomic features. Most of these features are non-coding RNAs or pseudo genes that have zero or very small number of RNA-seq read counts across most tumor samples. We selected 17,986 genes that have at least 20 reads in more than 25% of the samples for the down-stream analysis. Let *T*_*ij*_ be the read count for the *j*-th gene in the *i*-th sample. We correct for read-depth variation across samples using *T*_*ij*_/*d*_*i*_, where *d*_*i*_ is the 75 percentile of gene expression within the *i*-th sample, a robust measurement of read-depth [28]. Then we quantified gene expression by log(*T*_*ij*_/*d*_*i*_), to make variation of gene expression similar across orders of expression levels [29]. We further regressed out copy number effect from gene expression data (Section 3.4 in S3 Appendix). Since copy number measurement may be missing for some genes (usually the genes around the beginning/end of a chromosome or around a centromere), we removed genes with missing copy number information, and ended up with 16,339 genes for the following analysis.

Taking the intersection of the samples with somatic mutations and gene expression, we obtained 386 samples. We further included age, gender, and hyper-mutation status as covariates. We also removed those potential germline mutations by checking the read-depth data in the paired normal samples (Section 3.5 in S3 Appendix). In the following analysis, we only studied non-silent mutations because silent mutations in exonic regions are most likely to be passenger mutations that do not have functional impact.

#### mSAME results

For the mutation-level association analysis, we selected 37 mutations which have occurred in at least 5 of the 386 samples, corresponding to a mutation frequency of 1.3%. For the association analysis between these 37 mutations and all the 16,339 genes, the Bonferroni correction was adopted for multiple testing correction, i.e., we rejected the null hypothesis if the p-value was less than 0.05/(37 × 16339) ≈ 8.27 × 10^−8^.

We applied both mSAME and GLM to assess the associations between the somatic mutations and gene expression. Recall that we model alternative read count by a beta-binomial distribution, and the parameters of this distribution need to be estimated *a priori*. We estimated these parameters using the 3, 359 mutations used in gene-level analysis since more mutations can be included in gene-level analysis. In addition, the sensitivity and specificity for each mutation were estimated as described in Section 1.3 of S1 Appendix.

At the significance level with Bonferroni correction, mSAME identified 109 significant associations while GLM detected 100 significant associations that is a subset of the 109 associations identified by mSAME (Table S7 in S3 Appendix). Most of these significant associations (100 out of 109) are with respect to the BRAF V600E mutation (chr7:140753336), which is a single nucleotide variant that results in an amino acid change from a valine (V) to a glutamic acid (E). The high frequency of BRAF V600E mutation associations is partly due to its high mutation frequency (11.66%). In contrast, the secondly most frequently mutated locus, which is located in gene PIK3CA, is observed in only 3.63% of the samples (Fig S11 in S3 Appendix). Since this mutation has no detectable calling errors, the p-values of mSAME are in line with those of GLM in general.

In total, mSAME identified 9 additional findings that were missed by GLM. Here we briefly discuss two interesting examples and list all of them in Table 1. The first example is that the TP53 mutation “chr17:7673803” is associated with the gene expression CDX1. Previous work has indicated that the gene expression of CDX1 is abnormally down-regulated in colon cancer-derived cell lines [30, 31], and our finding suggests that this TP53 somatic mutation is partly responsible for dysregulation of CDX1’s expression in colon cancer. The second example is that TP53 mutation “chr17:7674894” associated with its own gene expression.

**Table 1:** Nine significant results detected uniquely by mSAME. An eQTL is a local eQTL if the distance between the somatic mutation and the gene is smaller than 1Mb. Otherwise it is a distant eQTL.

Mutation(Gene)	Associated Gene	eqtl type	mSAME	GLM
chr3:25627236(TOP2B)	C4orf19	distant	6.98e-8	8.64e-8
chr5:112838007(APC)	ZWILCH	distant	5.71e-9	8.58e-6
chr7:74824936(GTF2IRD2)	SDR42E1	distant	3.02e-10	1.30e-5
	LINC00675	distant	1.47e-8	1.02e-3
chr7:140753336(BRAF)	EPM2AIP1	distant	6.49e-8	8.44e-8
	LRRC19	distant	8.27e-8	9.72e-8
	ETV5	distant	6.91e-8	8.50e-8
chr17:7673803 (TP53)	CDX1	distant	1.77e-9	3.46e-2
chr17:7674894 (TP53)	TP53	local	3.30e-8	2.12e-7

#### gSAME results

We collapsed mutations within the same gene and obtained 17,386 gene-level mutations. Among these mutations, we conducted the association analysis for 180 genes that are mutated in at least 5 samples and are known to be associated with colon cancer. In total, these 180 gene-level mutations correspond to 3,359 individual mutations. We applied gSAME and GLM for all the 180 × 16, 339 tests, and uses Bonferroni corrected significance level of 1.70 × 10^−8^.

At this significance level, gSAME and GLM both identified 63 significant associations where 59 of them are in common, and hence 67 associations in total (Table S8 in S3 Appendix). Gene-level mutation status of BRAF is associated with the expression of 36 genes and all of these associations have been identified in mutation-level analysis with respect to the V600E mutation. This may not be surprising because V600E mutation is present in more than 80% of the samples with at least one BRAF mutation.

Another gene-level mutation that is associated with the expression of several genes is gene-level mutation of TP53. Each of the 68 individual mutations within TP53 has relatively low mutation frequency (the highest frequency is 5.70%), however, after aggregating all the mutations, the gene-level mutation TP53 is present in 39.38% of the samples. The expression of 11 genes are associated with TP53 gene-level mutation (including two gSAME-specific findings and one GLM-specific finding). Using the DAVID Tools for the gene enrichment analysis on these 11 genes (https://david.ncifcrf.gov/), we found that the following 4 genes are in the KEGG p53 signaling pathway: FAS, MDM2, DDB2, ZMAT3 (with enrichment p-value 3.1e-5 after Benjamini correction). Among them, FAS and ZMAT3 were only detected by gSAME (Table S8 in S3 Appendix). Intrigued by this functional enrichment, we further explored the gene-level associations for TP53 at a more liberal p-value cutoff of 0.05/16339 ≈ 3.06 × 10^−6^. gSAME and GLM both detected 27 associated genes, while 22 of them are in common. Among these 32 genes, the following seven genes belong to the KEGG p53 signaling pathway: BAX, FAS, MDM2, CDKN1A, DDB2, TP53I3, ZMAT3 (with enrichment p-value 3.3e-8 after Benjamini corretion), where BAX and TP53I3 are detected only by gSAME but missed by GLM.

The complete list of mutation level and gene level eQTL results can be found in two text files as in S1 and S2 Files.

### TCGA colon cancer subtype analysis

To illustrate somatic mutation association analysis using dichotomous outcomes, we applied both mSAME and gSAME to identify somatic mutations associated with colon cancer subtypes defined by DNA methylation data. One of the most well known subtype of colon cancer is the hypermutation subtype [4, 27]. By definition, it is associated with many somatic mutations and thus we used it as a covariate in all the analysis of this paper. Here we consider another subtype, defined by clustering analysis of genome-wide DNA methylation data [32] (Fig S13 in S3 Appendix). See Section 3.8 in S3 Appendix for details of methylation data processing. We used this clustering results to classify the cancer patients into two groups and treated it as a binary outcome. Then we associated this subtype indicator with somatic mutations.

Similar to the eQTL analysis, we performed mutation-level association analysis using mSAME and GLM (logistic regression) on 37 mutations that are present in at least 5 samples. At the significance level 0.05/37 ≈ 0.00135, mSAME and GLM both detected one significant mutation of BRAF V600E, where mSAME yields a smaller p-value than GLM (Table 2). We also performed gene-level analysis by gSAME and GLM for the 180 gene-level mutations used in eQTL analysis. Both methods discovered two significant gene-level mutations: BRAF and KMT2C, using the p-value threshold 0.05/180 = 0.00028. KMT2C is known as a tumor suppressor gene [33]. Our results suggest that the mutations of KMT2C are associated with DNA methylation, which is consistent with its role as histone methyltransferases because DNA methylation and histone methylation often work together to establish epigenetic landscape for gene expression regulation.

**Table 2:** Summary for the association study of the subtypes on mutation level (top table) and gene level (bottom table).

Mutation	Gene	mSAME	GLM
chr7:140753336	BRAF	5.91e-8	5.74e-6
**Mutation**	chr	gSAME	GLM
BRAF	7	4.41e-5	8.50e-5
KMT2C	7	2.44e-4	7.05e-4

### eQTL analysis for pan-cancer studies

Following the workflow of the eQTL analysis for TCGA Colon Adenocarcinoma (COAD) samples, we conducted eQTL analysis using somatic mutations for 11 other TCGA cancer types, including Bladder Urothelial Carcinoma (BLCA), Brain Lower Grade Glioma (LGG), Glioblastoma multiforme (GBM), Head and Neck squamous cell carcinoma (HNSC), Kidney renal clear cell carcinoma (KIRC), Liver Hepatocellular Carcinoma (LIHC), Lung adenocarcinoma (LUAD), Lung squamous cell carcinoma (LUSC), Ovarian serous cystadenocarcinoma (OV), Skin Cutaneous Melanoma (SKCM), and Stomach adenocarcinoma (STAD). These cancer types are chosen due to their relatively large sample sizes and relatively higher rates of somatic mutations.

We dowloaded the gene expression and somatic mutation data for association analysis from NCI GDC Data Portal, using the workflow of “HTSeq—Counts” for gene expression data, and the workflow of “MuTect2 Variant Aggregation and Masking” for somatic mutation data. For mutation-level association analysis, we selected the mutations that occur in at least 5 samples. For gene-level analysis, we selected the gene-level mutations that occur in at least 5% of the samples. For each mutated locus, we need read count data (read depth and the number of alternative reads) for all samples, regardless of mutation call status. For COAD analysis, we downloaded all the bam files to local server and then collected these counts. However, this approach is not feasible for pan-cancer study across 11 cancer types because downloading and storing all the bam files requires too many resources. Instead, we obtained the read-count data using the cloud service provided by The Seven Bridges Cancer Genomics Cloud [34] (Section 3.9 of S3 Appendix).

**Fig 3:**
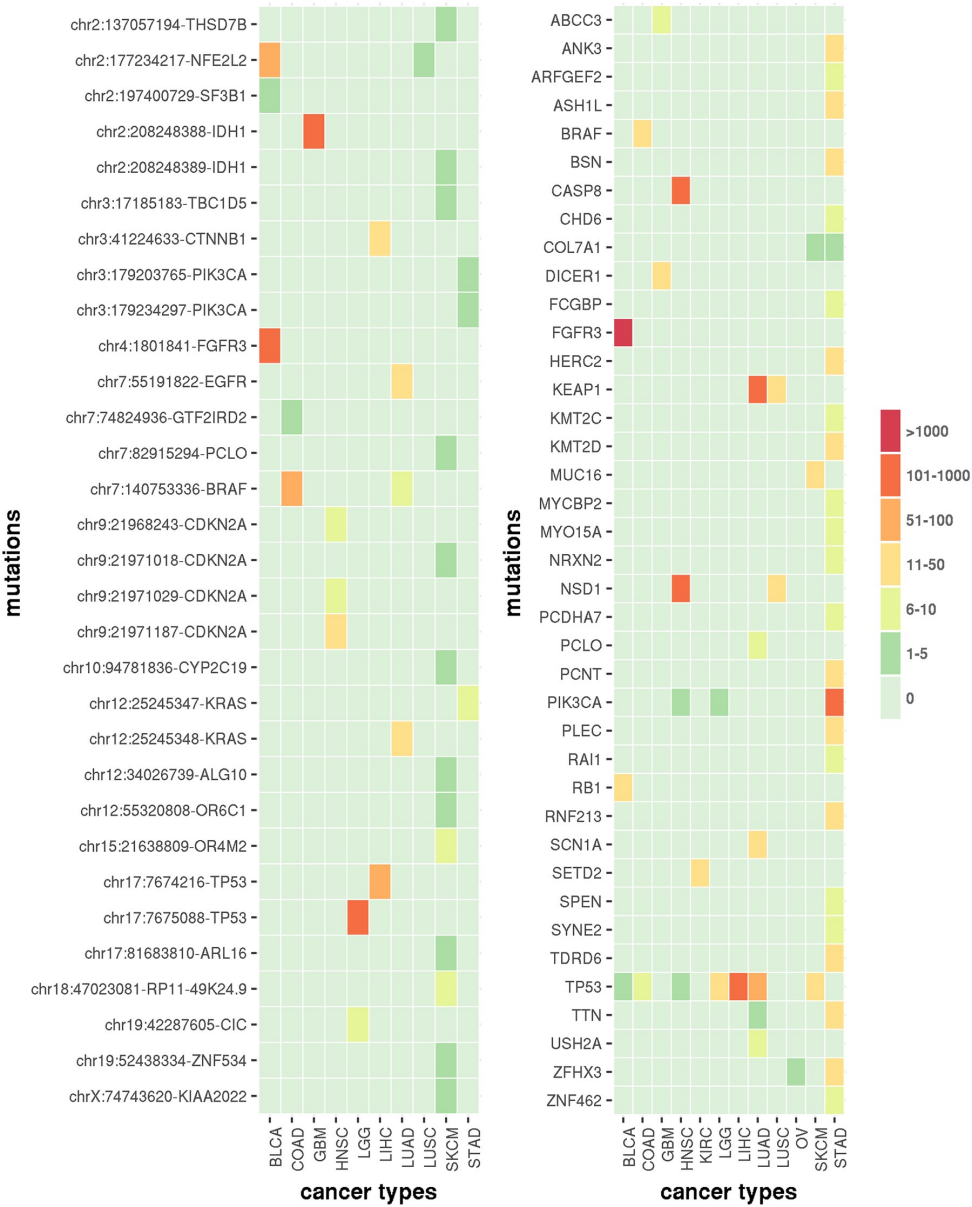
Summary of pan-cancer eQTL mapping results by SAME with Bonferroni multiple testing correction. Left panel: a heatmap of mutation-level eQTL mapping results by mSAME across cancer types. Each cell in the heatmap is colored according to the number of significant associations for one mutation (row) in one cancer type (column). Only those mutations that are associated with 2 or more genes across the 12 cancer types are shown. Note that OV cancer is not included since there is no significant eQTL in OV. Right panel: a heatmap of gene-level eQTL mapping results by gSAME across cancer types. Only those gene-level mutations that are associated with 6 or more genes across the 12 cancer types are shown.

In all association studies, we included age and gender (except for gender-specific cancer PRAD and OV) as covariates. For LGG, we further adjusted for cancer subtype defined based on the IDH1 or IDH2 mutation and chromosome 1p and 19q co-deletion [35]. We recorded significant findings using genome-wide Bonferroni correction, and summarized the number of the significant findings by GLM or SAME in Table S9 in S3 Appendix (Section 3.10 of S3 Appendix). The complete lists of the results are provided as supplementary text files. Examining the number of significant eQTLs for each mutation or each gene across cancer types shows no apparent pattern: most mutation-level or gene-level eQTLs are not shared across cancer types. However, one exception is gene-level TP53 mutation (Fig 3), which is among the significant eQTLs in 7 out of the 12 cancer types. This is partly due to the fact that TP53 is mutated with relatively high frequency across cancer types and it is a transcription factor that can directly regulate gene expression. When we relax the p-value cutoff to use transcriptome-wide significance (i.e., p-value cutoff = 0.05/# of genes), gene-level TP53 eQTLs were identified in 9 cancer types. In addition, several other gene-level eQTLs are shared among multiple cancer types (Fig S14 in S3 Appendix). Overall the pattern of mutation/gene eQTLs shared across cancer types are similar between SAME and GLM (Fig S15-S16 in S3 Appendix), though in general mSAME/gSAME identify more eQTLs than GLM.

**Fig 4:**
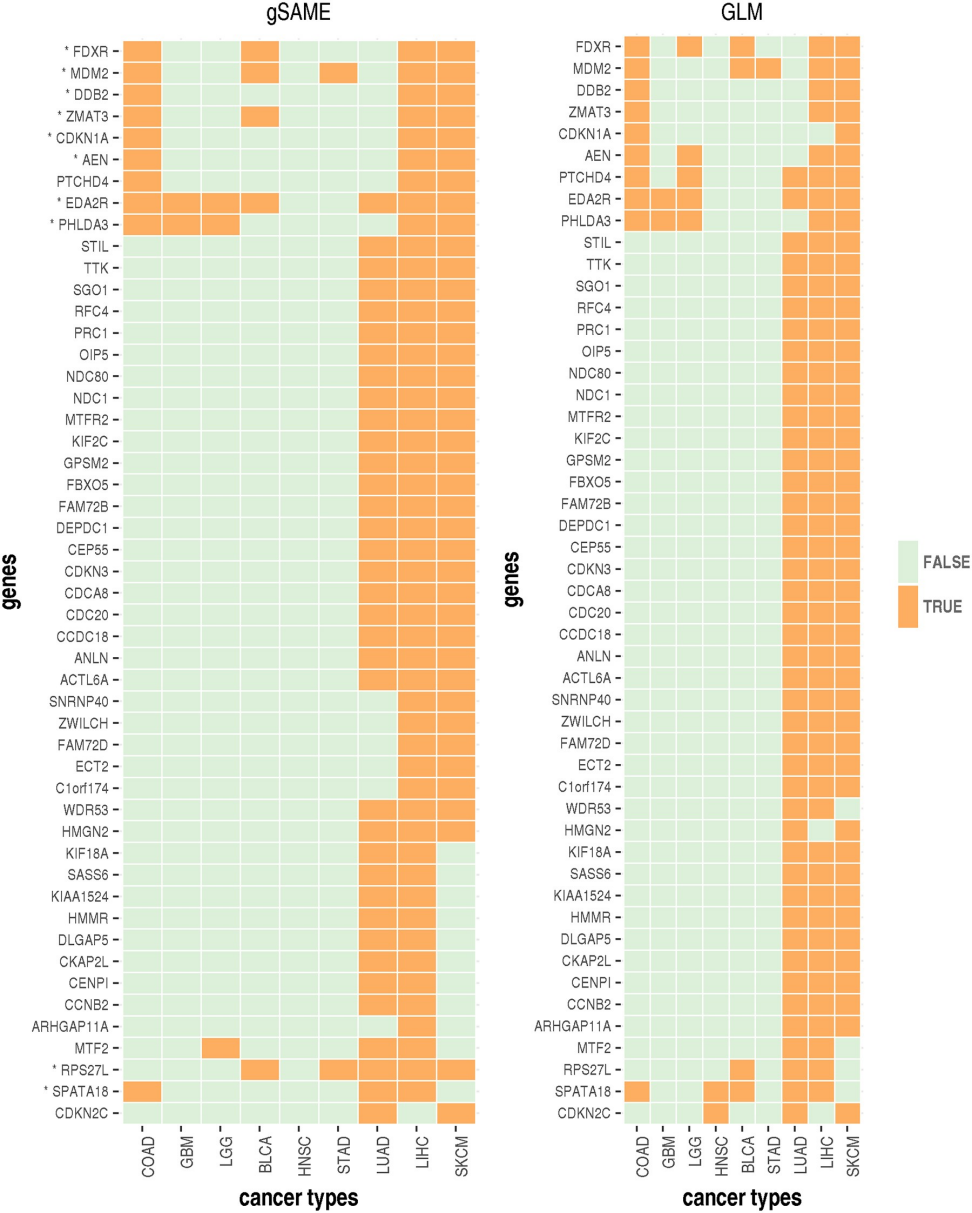
Summary of eGenes of TP53 with transcriptome-wide multiple testing correction. The results of gSAME (left panel) and GLM (right panel) were summarized by heatmaps showing whether a gene is eGene across cancer types. Only the genes that are eGenes for three or more cancer types by either method are shown. The asterisk (*) next to gene symbol indicates a gene is a high confidence TP53 target gene [37].

Next we examine the eGenes (genes whose expression are associated with an eQTL) associated with TP53 gene level mutation across cancer types. Since we focus on one mutation, we select the eGenes identified by transcriptome-wide significance. At this significance level, TP53 has no eGene by either gSAME or GLM in three cancer types: KIRC, LUSC and HNSC, and thus the following results only involve the remaining nine cancer types. We are interested in similarities of TP53 eGenes across cancer types. Towards this end, we examine the 50 eGenes identified in at least 3 cancer types by either gSAME (35 eGenes) or GLM (46 eGenes), with an intersection of 31 genes identified by both methods (Fig 4). The difference of gSAME and GLM results are most due to potential mutation calling errors in TP53 (Fig S17 in S3 Appendix).

The protein product of TP53, p53, is a very well studied tumor suppressor and is involved in different biological processes such as cell cycle arrest, DNA repair, and apoptosis [36]. Many target genes of p53 have been reported [37], and these target genes can be used to evaluate the relevance of the eGenes identified from our study. About 29% (10 out of 35) of the eGenes identified by gSAME and 20% (9 out of 46) identified by GLM are among 343 high confidence p53 target genes [37] (Fig 4). The only difference is gene CDKN1A (encoding protein p21) where gSAME and GLM identified it as an eGene for three and two cancer types, respectively. CDKN1A is one of the most important targets of p53 and is requested for p53-mediated cell cycle arrest [37].

Visualization of the mutation status of these 50 genes show an interesting pattern: three cancer types, LUAD, LIHC and SKCM are clustered together since many genes are eGenes only in these three cancer types (Fig 4). The relatively larger number of eGenes in these cancer types can not be explained by the mutation frequency of TP53 (Fig S18 in S3 Appendix) or genome-wide somatic mutation load (Fig S19 in S3 Appendix). None of these eGenes are among the 343 high confidence p53 target genes, suggesting that they may be indirectly regulated by p53. Gene ontology analysis shows that these eGenes are enriched with genes involved in cell cycle related biological processes such as chromosome segregation. Therefore our results suggest that somatic mutation of TP53 may have similar functional roles in cell cycle control in LUAD, LIHC and SKCM.

## Discussion

Understanding the associations between somatic mutations and cancer-related traits is of fundamental importance for precision cancer therapy. In this paper, we present a statistically powerful and computationally efficient approach for association analysis of somatic mutations while accounting for measurement errors of somatic mutations. By modeling the calling uncertainty of the somatic mutations and including the read-depth data into our statistical model, the proposed SAME method can significantly improve the statistical power for the association analysis. The SAME method can accommodate both continuous and dichotomous outcomes, and it is applicable to both mutation-level and gene-level association testing. While we have demonstrated SAME using the publicly available exome-seq data, it will provide larger degree of power gain for whole genome sequencing studies where read depth are typically lower.

**Fig 5:**
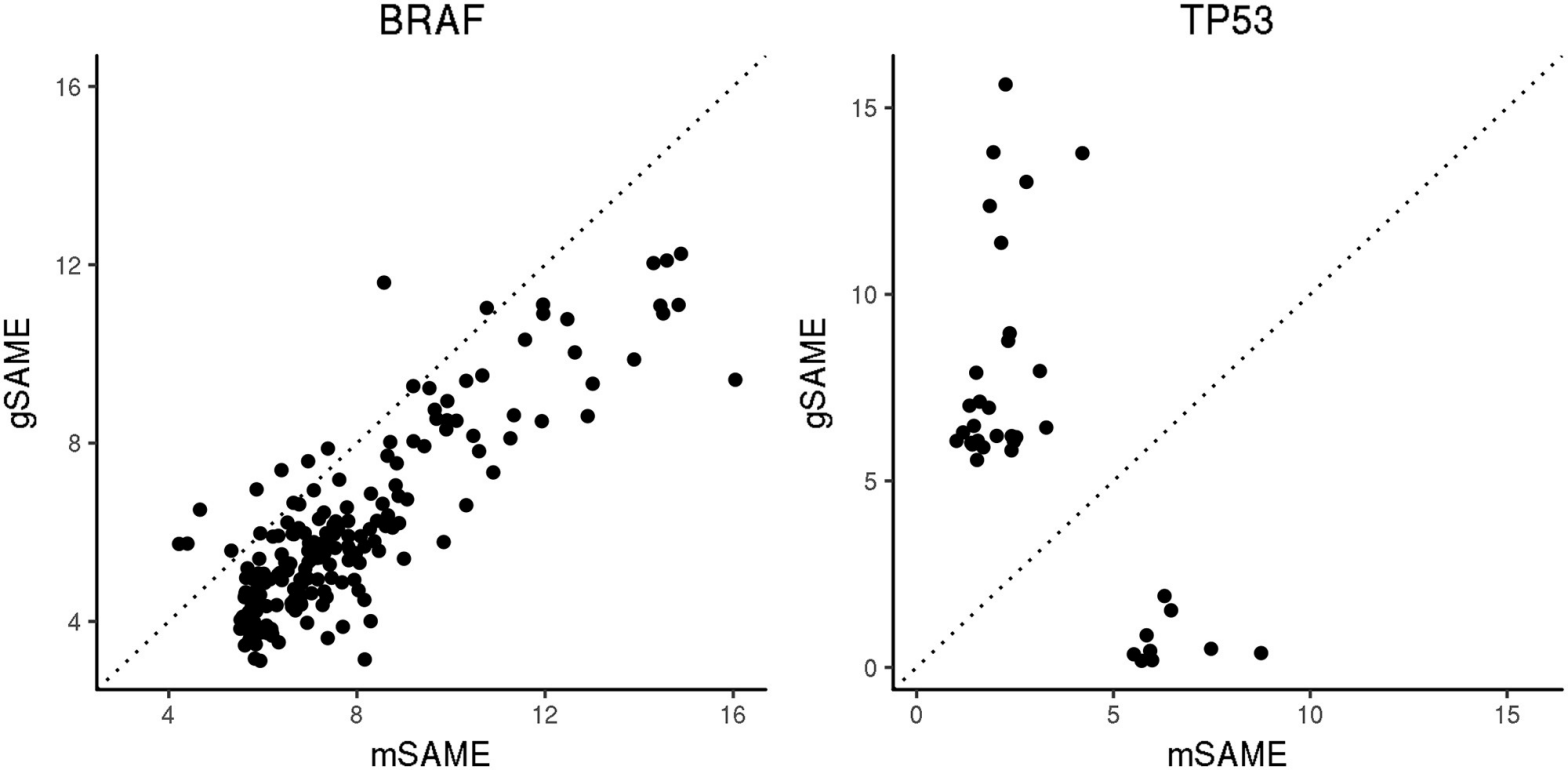
Comparison of eQTL mapping using mSAME p-values versus gSAME p-values on -log10 scale in colon cancer patients. Left panel: mutation-level versus gene-level analysis for the oncogene BRAF for all gene expression traits with eQTL p-value smaller than 0.05/16, 339 by either mSAME or gSAME. Right panel: similar results for the tumor suppressor gene TP53. Dashed line indicates *y* = *x*.

One practical question of using our method is that how to choose between mutation-level (mSAME) versus gene-level (gSAME) analysis. Our eQTL analysis results suggest that mSAME may be more suitable for recurrent mutations in oncogene (e.g., the BRAF V600E mutation). This is because an oncogene is often activated by some specific “gain of function” mutations that drive tumor growth, and such driver mutations are often recurrent across patients. Other rare mutations in the same gene may be passenger mutations, even if they are non-silent ones. For example, BRAF harbors 10 non-silent mutations in TCGA colon cancer dataset. Except for the V600E mutation, the remaining 9 mutations only occur in one or two samples, and thus are likely passenger mutations. When collapsing both driver and passenger mutations to create a gene-level mutation, the mutation pattern may be “diluted” by those passenger mutations, and thus gene-level associations may yield less significant results than mutation-level associations. This is indeed the pattern observed when we compare the eQTL results for BRAF V600E mutation versus BRAF gene level mutations (Fig 5).

In contrast, gSAME may be more suitable for tumor suppressor gene (e.g., TP53). The function of a tumor suppressor gene may be perturbed by multiple “loss of function” mutations and thus there is no evolutionary pressure to select a specific one. Since all the loss of function mutations have similar functional consequence, gene-level association can have much larger power than mutation-level analysis. For example, TP53 has 68 individual mutations in TCGA colon cancer dataset, among which only 6 mutations occur at more than 2% of the samples and are significant eQTLs with transcriptome-wide multiple testing correction. For each gene expression trait, we take the minimum mutation-level p-value across these 6 mutations and compare it with gene-level p-value. In most cases, the gene level analysis yields stronger associations than mutation-level analysis (Fig 5).

We have carefully implemented mSAME/gSAME to maximize computational efficiency, so that it is computationally feasible for genome-wide eQTL mapping. However, it still takes about 1-5 seconds per association testing. In contract, GLM is computationally much more efficient, taking about 0.01-0.02 seconds per association testing. Therefore, when there is limited mutation calling error (e.g., with high quality samples and high sequencing coverage) one strategy to balance computational time and accuracy is to use GLM for a quick initial scan, and then apply mSAME/gSAME for a subset of associations at a relatively liberal p-value cutoff. In addition, gSAME will become computationally more inefficient for larger analysis units, such as several genes within a pathway. Further development is needed in such situations. In fact, simply collapsing individual mutations may not be a good strategy for pathway level association analysis and better strategies to summarize pathway level somatic mutations warrant further studies.

Somatic mutation association is a new field with great potential to deliver key findings for precision cancer therapy. Accounting for somatic mutation calling uncertainty and low read-depth is an initial step to develop more rigorous and powerful association methods. We expect that more methods will be developed to exploit other types of information, such as intra-tumor heterogeneity or pathway level analysis where mutation information across genes is aggregated.

## Supporting information

S1 Appendix Supplementary methods. It includes details for (1) Estimation of beta-binomial distributions when read-depth is high; (2) Estimating the specificity and sensitivity of an individual somatic mutation; (3) Details of the EM algorithm for mSAME model; (4) Estimation of beta-binomial distributions when read-depth is low for gene-level associations; (5) Details of the EM algorithm for gSAME model.
(PDF)

S2 Appendix Additional simulation results. It includes the estimation error comparison of *β* by the EM algorithm and GLM, and additional simulations when the mutations calling becomes less accurate or the read-depth becomes lower compared with the simulations in the main text.
(PDF)

S3 Appendix Additional methods/results for real data analysis. It includes details for the data processing for the real data analysis: (1) Pre-processing and somatic mutation calling; (2) Mutation load and hypermutation status; (3) Allele-specific read counts; (4) Removing copy number effect from gene expression data; (5) Removing potential germline mutations; (6) Mutation frequencies for individual mutations or gene-level mutations; (7) Processing DNA methylation data.
(PDF)

S1 File The complete list of mutation level eQTL results. (TXT)

S2 File The complete list of gene level eQTL results. (TXT)
