# Correction: Association analysis using somatic mutations

**DOI:** 10.1371/journal.pgen.1007848

**Published:** 2018-12-06

**Authors:** Yang Liu, Qianchuan He, Wei Sun

The second author's name is spelled incorrectly. The correct name is: Qianchuan He.
