# Supplementary material for: Association analysis using somatic mutations
Source: PLoS Genet. 2018 Nov 2;14(11):e1007746. doi: 10.1371/journal.pgen.1007746 (PMC6235399; doi:10.1371/journal.pgen.1007746)
Supplement: S1 Appendix — It includes details for (1) Estimation of beta-binomial distributions when read-depth is high; (2) Estimating the specificity and sensitivity of an individual somatic mutation; (3) Details of the EM algorithm for mSAME model; (4) Estimation of beta-binomial distributions when read-depth is low for gene-level associations; (5) Details of the EM algorithm for gSAME model. (PDF) [file pgen.1007746.s001.pdf]

S1 Appendix. Supplementary Methods

1.1 Likelihood function for the mSAME model

For the conditional density $f_{A,D,O}(A_i, D_i, O_i|S_i)$ in the likelihood function of mSAME model, since O_i is missing when $D_i < d_0$, we can write it as

$$\begin{aligned} &f_D(D_i|S_i)[f_{A,O}(A_i, O_i|D_i, S_i)I(D_i \geq d_0) + f_A(A_i|D_i, S_i)I(D_i < d_0)] \\ =&f_D(D_i|S_i)[f_A(A_i|D_i, O_i, S_i)f_O(O_i|S_i)I(D_i \geq d_0) + f_A(A_i|D_i, S_i)I(D_i < d_0)], \end{aligned} \tag{1}$$

where $I(\cdot)$ is an indicator function. We ignore the distribution function $f_D(D_i|S_i)$ for hypothesis testing of $H_0 : \beta = 0$ because it does not involve any parameter of interest, and thus can be considered as a constant. When $D_i \geq d_0$, we write $f_A(A_i|D_i, O_i, S_i)$ as a beta-binomial distribution that depends on the observed mutation call O_i and the underlying mutation status S_i:

$$f_A(A_i|D_i, O_i, S_i) = \begin{cases} f_{bb}(A_i|D_i, \pi_{00}, \varphi_{00}) & \text{if } O_i = 0, S_i = 0, \\ f_{bb}(A_i|D_i, \pi_{01}, \varphi_{01}) & \text{if } O_i = 0, S_i = 1, \\ f_{bb}(A_i|D_i, \pi_{10}, \varphi_{10}) & \text{if } O_i = 1, S_i = 0, \\ f_{bb}(A_i|D_i, \pi_{11}, \varphi_{11}) & \text{if } O_i = 1, S_i = 1, \end{cases} \tag{2}$$

where f_{bb} denotes a beta-binomial distribution, $\pi_{00}, \pi_{01}, \pi_{10}, \pi_{11}$ and $\varphi_{00}, \varphi_{01}, \varphi_{10}, \varphi_{11}$ are the corresponding mean and over-dispersion parameters for the beta-binomial distributions. We model $f_O(O_i|S_i)$ as a Bernoulli distribution that depends on S_i:

$$f_O(O_i|S_i) = \begin{cases} f_{ber}(O_i, 1 - \gamma_0) & \text{if } S_i = 0, \\ f_{ber}(O_i, \gamma_1) & \text{if } S_i = 1, \end{cases} \tag{3}$$

where γ_0 and γ_1 are the specificity (1 - false positive rate) and sensitivity (1 - false negative rate) of the somatic mutation calls, respectively.

When $D_i < d_0$, we do not observe O_i and we write $f_A(A_i|D_i, S_i)$ as

$$f_A(A_i|D_i, S_i) = \begin{cases} f_{bb}(A_i|D_i, \pi_0, \varphi_0) & \text{if } S_i = 0, \\ f_{bb}(A_i|D_i, \pi_1, \varphi_1) & \text{if } S_i = 1. \end{cases} \tag{4}$$

To check the validity of the model for the read-depth data in equation (2), we collect the read-depth data from those 37 mutations we analyzed in the mutation level analysis for

TCGA COAD data. For the read-depth data for which mutation is not called (O = 0), we fit a mixture of two beta-binomial distributions: 0.9836 f_{bb}(0.0011, 0.0005) + 0.0164 f_{bb}(0.0327, 0.3332). Similarly we fit a mixture of two beta-binomial distributions for the read-depth data for which mutation is called (O = 1): 0.0053 f_{bb}(0.0518, 0.0001) + 0.9947 f_{bb}(0.1552, 0.0053). Based on these two models, we can generate the numbers of alternative reads given observed the read-depths. In order to evaluate how good these two models fit the real data, we check the QQ plots and apply the Kolmogorov-Smirnov test for the generated numbers of alternative reads and the observed numbers of alternative reads. As seen in Fig S1, these two QQ plots show that the distributions of generated data and the observed data agree very well. This conclusion is also confirmed by comparing the expected and observed read counts using the Kolmogorov-Smirnov test, which gives p-values of 0.99 and 0.31 for O=0 and O=1, respectively. These results demonstrate that beta-binomial distribution could provide a good fit for the real data in our study.

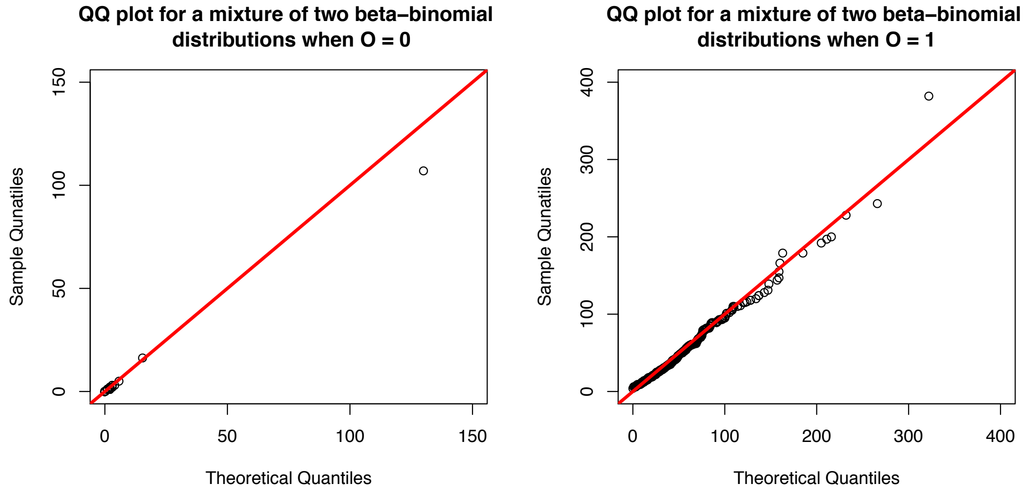

Fig S1: QQ plots to compare the distribution of observed alternative read count distribution (y-axis) versus expected distribution based on model fit (x-axis) when somatic mutation is called (O=1) or not (O=0). When O = 0, 87.10% of the alternative read counts based on model fit and 87.19% of the observed read counts are zeros, therefore most of quantiles are very small in the left panel.

1.2 Estimation of beta-binomial distributions when read-depth is high

In practice, there is limited information to estimate the parameters in those four beta-binomials in equation (2): $\pi_{00}, \pi_{01}, \pi_{10}, \pi_{11}$ and $\varphi_{00}, \varphi_{01}, \varphi_{10}, \varphi_{11}$ for each mutation separately. It is reasonable to assume these parameters are shared across mutations. For example, a true mutation tend to have larger variant allele frequency (VAF), hence larger π, than a false positive. Therefore we estimate these eight parameters by pooling the data across all genes and all samples.

Using the read-depth data for which mutation is not called ($O = 0$), we fit a mixture of two beta-binomial distributions, and estimate π_{00}, φ_{00} by the parameters of the beta-binomial with smaller mean, and π_{01}, φ_{01} by the parameters of the other beta-binomial. Analogously, we fit a mixture of two beta-binomial distributions using the read-depth data for which mutations are called ($O = 1$), and then estimate π_{10}, φ_{10} by the parameters of the beta-binomial with smaller mean, and π_{11}, φ_{11} by the parameters of the other beta-binomial.

1.3 Estimating the sensitivity and specificity of an individual somatic mutation

For either mSAME or gSAME model, we assume that the sensitivity and specificity of mutation calls for each somatic mutation are known. They can be estimated using external data of systematic validation studies, or alternatively, using the observed mutation calls and read count data, and we will describe the latter approach in the following.

To estimate sensitivity or specificity, we need to infer the underlying mutation status of the mutation of interest across all samples, denoted by $\hat{S}_i$. Then sensitivity can be calculated as the proportion of samples where mutations are called among those with estimated mutation status being 1: $\sum_{i=1}^n O_i\hat{S}_i / \sum_{i=1}^n \hat{S}_i$. Similarly, specificity can be estimated as $\sum_{i=1}^n (1-O_i)(1-\hat{S}_i) / \sum_{i=1}^n (1-\hat{S}_i)$. This may appear to be a very challenging task since it requires the estimation of $\hat{S}_i$'s. However, since what we want to estimate is an average across n samples, the result is robust to potential estimate bias of individual $\hat{S}_i$'s. Next we describe how to obtain $\hat{S}_i$'s.

We model the number of alternative reads (A_i) given read depth (D_i) by a mixture of two beta-binomial distributions: $\rho_0' f_{bb}(A_i|D_i; \pi_0', \varphi_0') + (1 - \rho_0') f_{bb}(A_i|D_i; \pi_1', \varphi_1')$, where $f_{bb}(A_i|D_i; \pi_0', \varphi_0')$ and $f_{bb}(A_i|D_i; \pi_1', \varphi_1')$ correspond to the cases with the underlying mutation status being 0 and 1, respectively. Intuitively, if there is no mutation, the mean value of the beta-binomial distribution (π_0'), which is an estimate of the mutant allele frequency A_i/D_i, should be small. Let $p_{i0} = \rho_0' f_{bb}(A_i|D_i; \pi_0', \varphi_0')$ and $p_{i1} = (1-\rho_0') f_{bb}(A_i|D_i; \pi_1', \varphi_1')$, then $\hat{S}_i$ can be estimated as 0 if $p_{i1}/(p_{i1} + p_{i0}) < 0.5$, and as 1 otherwise.

A somatic mutation may only occur in a few samples, and thus it is highly unstable to estimate this mixture distribution using the data from one mutation across n samples. Instead, we used a two-step approach. We first estimate $f_{bb}(A_i|D_i; \pi_0', \varphi_0')$ and $f_{bb}(A_i|D_i; \pi_1', \varphi_1')$ by pooling information across all mutations. Specifically, we estimate $f_{bb}(A_i|D_i; \pi_1', \varphi_1')$ using all the cases (across all the mutations and all the samples) where mutations are called, and estimate $f_{bb}(A_i|D_i; \pi_0', \varphi_0')$ using all the cases where mutations are not called. Of course this procedure has an implicit assumption that the observed mutation calls are overall consistent with underlying mutation status, and and thus we can estimate $f_{bb}(A_i|D_i; \pi_0', \varphi_0')$ and $f_{bb}(A_i|D_i; \pi_1', \varphi_1')$ based on observed mutation calls. This assumption may be invalid for some mutations and thus leads to estimation bias. However, when we pool information across all mutations to estimate the densities, such bias is unlikely to have large impact. Those cases with low read-depth are not included in the estimation process because the mutation call status is ambiguous. In the second step or our estimation procedure, we estimate a mutation-specific mixture proportion ρ_0', because the mutation frequency can vary from a few percentages to more than 50% across mutations.

1.4 Estimation of the other parameters using EM algorithm

Denote the unknown parameters of the likelihood by

$$\theta = (\rho_0, \alpha, \beta, \phi, \pi_0, \varphi_0, \pi_1, \varphi_1),$$

we can re-write the likelihood of the observed data as

$$\mathcal{L}(\theta; Y, A, D, O) = \prod_{i=1}^{n} \sum_{j=0}^{1} \rho_j f_{Y,A,D,O}(Y_i, A_i, D_i, O_i | S_i = j; \theta). \tag{5}$$

Direct optimization for the likelihood is difficult due to the existence of the latent variable S_i. Hence we use the following EM algorithm to find the maximum likelihood parameters of the model. The complete likelihood can be written as

$$\mathcal{L}(\theta; Y, A, D, O, S) = \prod_{i=1}^{n} \sum_{j=0}^{1} \mathbf{I}(S_i = j) \rho_j f_{Y,A,D,O}(Y_i, A_i, D_i, O_i | S_i = j; \theta). \tag{6}$$

Given the current estimate of the parameter

$$\theta^{(t)} = (\rho_0^{(t)}, \alpha^{(t)}, \beta^{(t)}, \phi^{(t)}, \pi_0^{(t)}, \varphi_0^{(t)}, \pi_1^{(t)}, \varphi_1^{(t)}),$$

we compute the conditional distribution of S_i conditional on the observed data as

$$\begin{aligned} \eta_{ji}^{(t)} &= P(S_i = j | Y, X, A, D, O; \theta^{(t)}) \\ &= \frac{\rho_j^{(t)} f_{Y,A,D,O}(Y_i, A_i, D_i, O_i | S_i = j; \theta^{(t)})}{\sum_{j=0}^{1} \rho_j^{(t)} f_{Y,A,D,O}(Y_i, A_i, D_i, O_i | S_i = j; \theta^{(t)})}. \end{aligned} \tag{7}$$

The E-step corresponds with setting up the following Q function:

$$\begin{aligned} Q(\theta|\theta^{(t)}) =& E_{S_i|Y,X,A,D,O;\theta^{(t)}} \log \mathcal{L}(\theta; Y, X, A, D, O, S) \\ =& \sum_{i=1}^{n} \sum_{j=0}^{1} \eta_{ji}^{(t)} \log f_{Y,A,D,O}(Y_i, A_i, D_i, O_i | S_i = j; \theta^{(t)}) \\ =& \sum_{i=1}^{n} \sum_{j=0}^{1} \eta_{ji}^{(t)} \left[\log \rho_j + \log f_Y(Y_i | S_i = j)\right] + \sum_{i:D_i \geq d_0} \sum_{j=0}^{1} \eta_{ji}^{(t)} \log f_{A,D,O}(A_i, D_i, O_i | S_i = j) \\ &+ \sum_{i:D_i < d_0} \sum_{j=0}^{1} \eta_{ji}^{(t)} (\log f_A(A_i | D_i; \pi_j, \varphi_j) + \log f_D(D_i | S_i = j)). \end{aligned} \tag{8}$$

We can consider the maximization over multiple subsets of θ seperately since the parameters appear in separate linear terms in $Q(\theta|\theta^{(t)})$.

For ρ_0, we have

$$\begin{aligned}\rho_0^{(t+1)} &= \underset{\rho_0}{\text{argmax}} Q(\theta|\theta^{(t)}) \\ &= \underset{\rho_0}{\text{argmax}} \left[\sum_{i=1}^n \eta_{0i}^{(t)} \log \rho_0 + \sum_{i=1}^n \eta_{1i}^{(t)} \log(1-\rho_0)\right] \\ &= \frac{1}{n}\sum_{i=1}^n \eta_{0i}^{(t)},\end{aligned} \tag{9}$$

and $\rho_1^{(t+1)} = 1 - \rho_0^{(t+1)}$.

For α, β, ϕ, we have for continuous trait:

$$\begin{aligned}(\alpha^{(t+1)}, \beta^{(t+1)}, \phi^{(t+1)}) = \underset{\alpha,\beta,\phi}{\text{argmax}} &\left[\sum_{i=1}^n \eta_{0i}^{(t)} \left(-\frac{1}{2}\log\phi - \frac{(Y_i - x_i^T\alpha)^2}{2\phi}\right) + \right. \\ &\left. \sum_{i=1}^n \eta_{1i}^{(t)} \left(-\frac{1}{2}\log\phi - \frac{(Y_i - x_i^T\alpha - \beta)^2}{2\phi}\right)\right],\end{aligned}$$

which yields

$$\alpha^{(t+1)} = \left(X^TMX\right)^{-1} X^TMY, \tag{10}$$

$$\beta^{(t+1)} = \frac{(H_1^{(t)})^T(Y - X^T\alpha^{(t+1)})}{\sum_{i=1}^n \eta_{1i}^{(t)}}, \tag{11}$$

$$\phi^{(t+1)} = \sum_{i=1}^n \frac{\eta_{0i}^{(t)}(Y_i - x_i^T\alpha^{(t+1)})^2}{n} + \sum_{i=1}^n \frac{\eta_{1i}^{(t)}(Y_i - x_i^T\alpha^{(t+1)} - \beta^{(t+1)})^2}{n}, \tag{12}$$

where $M = H_1^{(t)}(H_1^{(t)})^T - \sum_{i=1}^n \eta_{1i}^{(t)} I_n$ with $H_1^{(t)} = (\eta_{11}^{(t)}, \cdots, \eta_{1n}^{(t)})^T$.

For binary traits, we always have $\phi = \phi^{(t)} = \phi^{(t+1)} = 1$, and

$$\begin{aligned}(\alpha^{(t+1)}, \beta^{(t+1)}) = \underset{\alpha,\beta}{\text{argmax}} &\left[\sum_{i=1}^n \eta_{0i}^{(t)} \left(Y_i x_i^T\alpha - \log(1+\exp(x_i^T\alpha))\right) + \right. \\ &\left. \sum_{i=1}^n \eta_{1i}^{(t)} \left(Y_i x_i^T\alpha + Y_i\beta - \log(1+\exp(x_i^T\alpha+\beta))\right)\right].\end{aligned}$$

The above optimization problem can be solved by the following Fisher scoring algorithm,

$$\begin{bmatrix}\alpha_{m+1}\\ \beta_{m+1}\end{bmatrix} = \begin{bmatrix}\alpha_m\\ \beta_m\end{bmatrix} + \begin{bmatrix}X^T(D_0'(m)+D_1'(m))X & X^TD_1'(m)1_n \\ 1_n^TD_1'(m)X & 1_n^TD_1'(m)1_n\end{bmatrix}^{-1} \begin{bmatrix}X^T(Y - D_0(m) - D_1(m)) \\ (H_1^{(t)})^TY - D_1(m)1_n\end{bmatrix}, \tag{13}$$

where $1_n = (1, \cdots, 1)^T$, $H_1^{(t)} = (\eta_{11}^{(t)}, \cdots, \eta_{1n}^{(t)})^T$ and

$$\begin{aligned}
D_0'(m) &= diag\left(\frac{\eta_{01}^{(t)}\exp(x_1^T\alpha_m)}{[1+\exp(x_1^T\alpha_m)]^2}, \cdots, \frac{\eta_{0n}^{(t)}\exp(x_n^T\alpha_m)}{[1+\exp(x_n^T\alpha_m)]^2}\right), \\
D_1'(m) &= diag\left(\frac{\eta_{11}^{(t)}\exp(x_1^T\alpha_m+\beta_m)}{[1+\exp(x_1^T\alpha_m+\beta_m)]^2}, \cdots, \frac{\eta_{1n}^{(t)}\exp(x_n^T\alpha_m+\beta_m)}{[1+\exp(x_n^T\alpha_m+\beta_m)]^2}\right), \\
D_0(m) &= diag\left(\frac{\eta_{01}^{(t)}\exp(x_1^T\alpha_m)}{1+\exp(x_1^T\alpha_m)}, \cdots, \frac{\eta_{0n}^{(t)}\exp(x_n^T\alpha_m)}{1+\exp(x_n^T\alpha_m)}\right), \\
D_1(m) &= diag\left(\frac{\eta_{11}^{(t)}\exp(x_1^T\alpha_m+\beta_m)}{1+\exp(x_1^T\alpha_m+\beta_m)}, \cdots, \frac{\eta_{1n}^{(t)}\exp(x_n^T\alpha_m+\beta_m)}{1+\exp(x_n^T\alpha_m+\beta_m)}\right).
\end{aligned}$$

For $\pi_0, \varphi_0, \pi_1, \varphi_1$, we have

$$(\pi_0^{(t+1)}, \varphi_0^{(t+1)}) = \underset{\pi_0,\varphi_0}{\text{argmax}} \sum_{i:D_i<d_0} \eta_{0i}^{(t)} \log f_A(A_i|D_i; \pi_0, \varphi_0), \quad (14)$$

$$(\pi_1^{(t+1)}, \varphi_1^{(t+1)}) = \underset{\pi_1,\varphi_1}{\text{argmax}} \sum_{i:D_i<d_0} \eta_{1i}^{(t)} \log f_A(A_i|D_i; \pi_1, \varphi_1), \quad (15)$$

which can be solved by numeric algorithms.

The iteration process of E-step and M-step will be terminated if $||\theta^{(t+1)} - \theta^{(t)}||_\infty < \epsilon$ for some preset threshold ϵ. We set the initial values of the parameters as $\rho_0^{(0)} = 0.5, \beta^{(0)} = 0, \pi_0^{(0)} = 0.001, \varphi_0^{(0)} = 0.0054, \pi_1^{(0)} = 0.15, \varphi_1^{(0)} = 0.10$, and $\alpha^{(0)}$ and $\phi^{(0)}$ to be the maximum likelihood estimators under the null model: $E(Y_i) = g^{-1}(x_i^T\alpha)$.

1.5 Likelihood function for the gSAME model

For the i-th sample, when the gene-level mutation status $S_i^g = 1$, there are 2^{m_i} possible combinations for the mutation-level mutation status S_i^m. Recall that m_i is the number of mutations with at least one alternative read (i.e., $A_{ij}^m > 0$). Particularly, for the k-th combination where the true mutation values are $s_i^{(k)} = \{s_{i1}^{(k)}, \cdots, s_{ip}^{(k)}\}$, $k = 1, \cdots, 2^{m_i} - 1$, we have

$$\delta_{ik} \triangleq P(S_i^m = s_i^{(k)}) = \prod_{j=1}^{p} w_j^{s_{ij}^{(k)}} (1 - w_j)^{1-s_{ij}^{(k)}}, \quad (16)$$

where $w_j = P(S_{ij}^m = 1)$, which can be estimated by the observed frequency of the j-th mutation across the n samples, or in an external reference population if such data are available. Then

we have

$$\delta_{ik}^* \triangleq P(S_i^m = s_i^{(k)} | S_i^g = 1) = \frac{P(S_i^m = s_i^{(k)}, S_i^g = 1)}{\sum_{l=1}^{2^{m_i}-1} P(S_i^m = s_i^{(l)})} = \frac{\delta_{ik}}{\sum_{l=1}^{2^{m_i}-1} \delta_{il}}. \tag{17}$$

Given the above modeling of $P(S_i^m = s_i^{(k)} | S_i^g = 1)$, and let $\rho_0^g = P(S_i^g = 0)$, then we can write out the likelihood for the i-th sample as the summation of two terms: $l_i = \rho_0^g l_{i0} + (1 - \rho_0^g) l_{i1}$, where l_{i0} and l_{i1} denote the likelihood when $S_i^g = 0$ or $S_i^g = 1$, respectively. More specifically,

$$\begin{aligned} l_{i0} &= f_{Y,A,D,O}(Y_i, A_i^m, D_i^m, O_i^m | S_i^g = 0) \\ &= f_Y(Y_i | S_i^g = 0) P(S_i^m = \mathbf{0} | S_i^g = 0) f_{A,D,O}(A_i^m, D_i^m, O_i^m | S_i^m = \mathbf{0}) \\ &= f_Y(Y_i | S_i^g = 0) \prod_{j=1}^{p} f_{A,D,O}(A_{ij}^m, D_{ij}^m, O_{ij}^m | S_{ij}^m = 0), \end{aligned} \tag{18}$$

and

$$\begin{aligned} l_{i1} &= f_{Y,A,D,O}(Y_i, A_i^m, D_i^m, O_i^m | S_i^g = 1) \\ &= f_Y(Y_i | S_i^g = 1) \sum_{k=1}^{2^{m_i}-1} P(S_i^m = s_i^{(k)} | S_i^g = 1) f_{A,D,O}(A_i^m, D_i^m, O_i^m | S_i^m = s_i^{(k)}) \\ &= f_Y(Y_i | S_i^g = 1) \sum_{k=1}^{2^{m_i}-1} \delta_{ik}^* \prod_{j=1}^{p} f_{A,D,O}(A_{ij}^m, D_{ij}^m, O_{ij}^m | S_{ij}^m = s_{ij}^{(k)}). \end{aligned} \tag{19}$$

As in the case for mutation-level analysis, we denote $f_Y(Y_i | S_i^g)$ through a generalized linear model with the mean $g^{-1}(x_i^T \alpha + S_i^g \beta)$ and a dispersion parameter ϕ, and $f_{A,D,O}(A_{ij}^m, D_{ij}^m, O_{ij}^m | S_{ij}^m)$ is the conditional density of A_{ij}, D_{ij}, O_{ij} for a single mutation, which has been defined in equation (1).

1.6 Parameter estimation for the gSAME model

Similar to the mutation level analysis, the parameters $\pi_{00}, \pi_{01}, \pi_{10}, \pi_{11}$, $\varphi_{00}, \varphi_{01}, \varphi_{10}, \varphi_{11}$, and the specificity γ_{0j}, the sensitivity γ_{1j} for the jth mutation are pre-specified or estimated. Since there are more low read-depth data in the gene-level analysis, we can pre-estimate the parameters π_0, π_1, and φ_0, φ_1 by borrowing information across all mutations within a gene to reduce the model complexity. Specifically, for each gene, we pool all the low-read-depth data

across all samples and all mutations within the gene together, and then fit a mixture of two beta-binomial distributions. The parameters π_0, φ_0 are estimated by the parameters of the beta-binomial with smaller mean, and π_1, φ_1 by the parameters of the other beta-binomial.

We use an EM algorithm to estimate the remaining parameters. Given the current estimator

$$\theta^{(t)} = (\rho_0^{g(t)}, \alpha^{(t)}, \beta^{(t)}, \phi^{(t)}),$$

the density of S_i^g conditional on the observed data is

$$\begin{aligned} \eta_{0i}^{(t)} &= P(S_i^g = 0 | Y_i, A_i^m, D_i^m, O_i^m; \theta^{(t)}) \\ &= \frac{\rho_0^{g(t)} f_{Y,A,D,O}(Y_i, A_i^m, D_i^m, O_i^m | S_i^g = 0; \theta^{(t)})}{\sum_{l=0}^{1} \rho_l^{g(t)} f_{Y,A,D,O}(Y_i, A_i^m, D_i^m, O_i^m | S_i^g = l; \theta^{(t)})} \end{aligned} \tag{20}$$

Analogously, the parameters $\rho_0^g, \alpha, \beta, \phi$ could be updated using (9) - (13). For the parameters under H_0, we estimate α, ϕ directly using the maximum likelihood estimator under the null model and only update ρ_0^g using (9) in the EM algorithm.
