# Supplementary material for: Association analysis using somatic mutations
Source: PLoS Genet. 2018 Nov 2;14(11):e1007746. doi: 10.1371/journal.pgen.1007746 (PMC6235399; doi:10.1371/journal.pgen.1007746)
Supplement: S2 Appendix — It includes the estimation error comparison of β by the EM algorithm and GLM, and additional simulations when the mutations calling becomes less accurate or the read-depth becomes lower compared with the simulations in the main text. (PDF) [file pgen.1007746.s002.pdf]

S2 Appendix. Additional simulation results

We provided the estimation error of β via the EM algorithm in the section. Under the same settings in the simulations, we set $\beta = 1$ for the continuous trait and $\beta = 2$ for the binary trait and compared the mean squared error (MSE) for SAME and GLM. For each setup, we record the median of the MSEs over 1000 replicates. The results of the mean squared error (MSE) for both mutation-level and gene-level simulations were displayed in Fig S2. It is observed that SAME provide more accurate estimators than those of GLM under all settings.

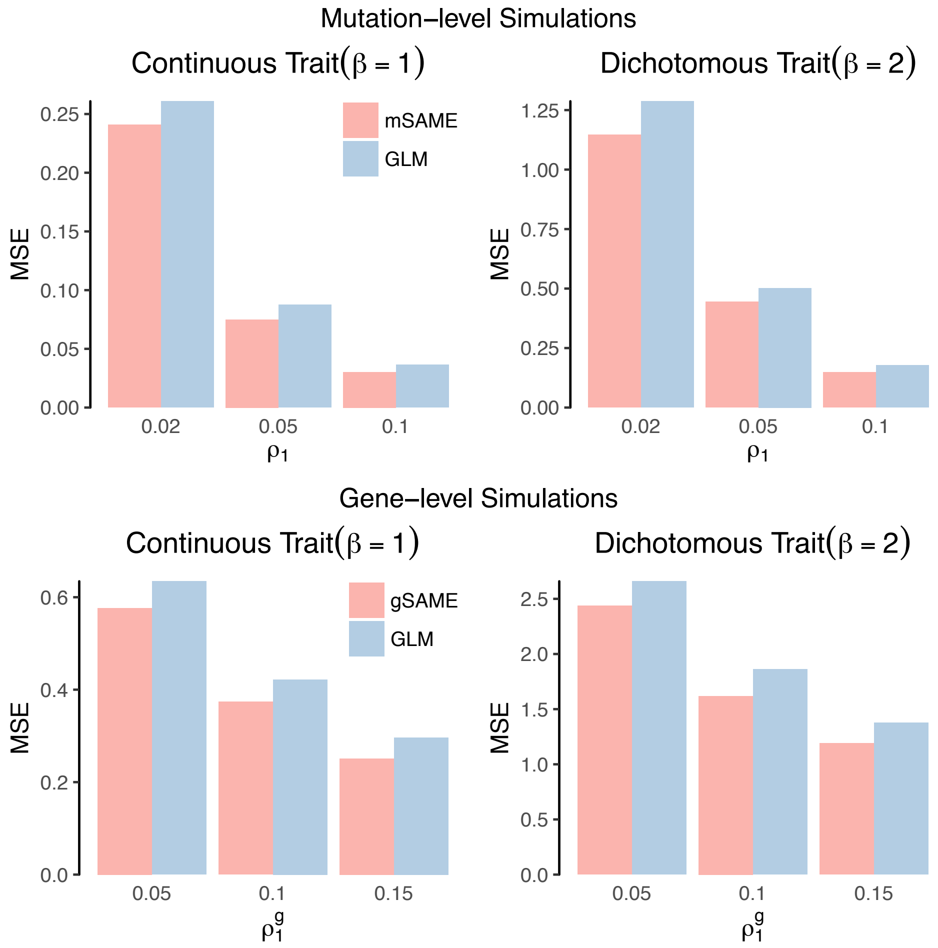

Fig S2: Mean squared errors of SAME and GLM for the mutation-level simulations (Top Panel) and the gene-level simulations (Bottom Panel).

We also conducted additional simulations for the situations when the mutations calling becomes less accurate or the read-depth becomes lower compared with the simulations in the

main text. Specifically, we consider the following two scenarios:

scenario (1) the specificity of mutation calls is set to be 0.95;

scenario (2) the read-depth data is simulated by a negative binomial distribution with mean 40 and over-dispersion 1.9.

For each scenario, the remaining settings of simulations are the same as in the main text. The power performance under these two scenarios are displayed in Figure S5 - Figure S8 (also see Table S3 - S6). It can be seen that the power gain of the proposed mSAME and gSAME over GLM in these two scenarios is comparable to or larger than that of the simulations in the main text.

In Figure S4, we further compared the power ratio of mSAME over GLM for the continuous trait with $\rho_1 = 0.05$, and the power ratio of gSAME over GLM for the continuous trait with $\rho_1 = 0.10$ for the simulation setup in the main text (default), scenario (1), and scenario (2). The power ratios of scenario (1) and scenario (2) are larger than that of the default simulations in most cases. We notice that in scenario (2), on average about 27.32% of the samples have read depth lower than 20. For the simulations in the main text, since average read-depth varies across genes and thus expected number of samples with read-depth lower than 20 varies across genes (See Figure S3). For most of the genes, this proportion is small. In fact, this proportion is smaller than 27.32% for more than 90% of the genes.

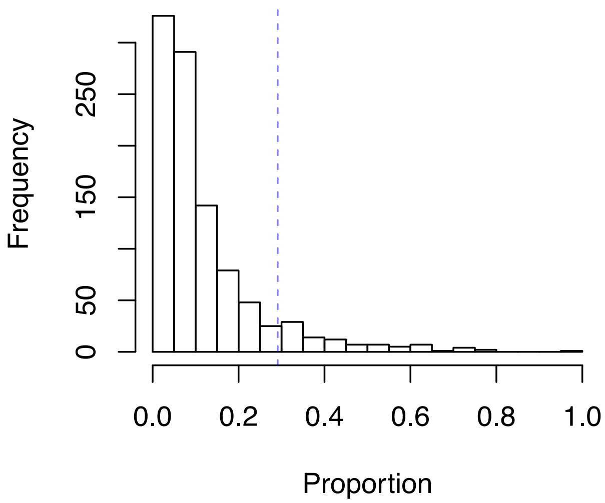

Fig S3: Histogram of the expected proportions of samples with read depth lower than 20 across all mutations for the simulation setup in main text that resembles an exome-seq dataset. Dashed line represents the expected proportion of samples with read depth lower than 20 in scenario (2), which mimic a whole-genome sequencing dataset. We need to summarize the proportion of samples with low coverage in exome-seq simulations by a histogram because read-depth of each mutation has its own mean values, and thus its own expected proportion.

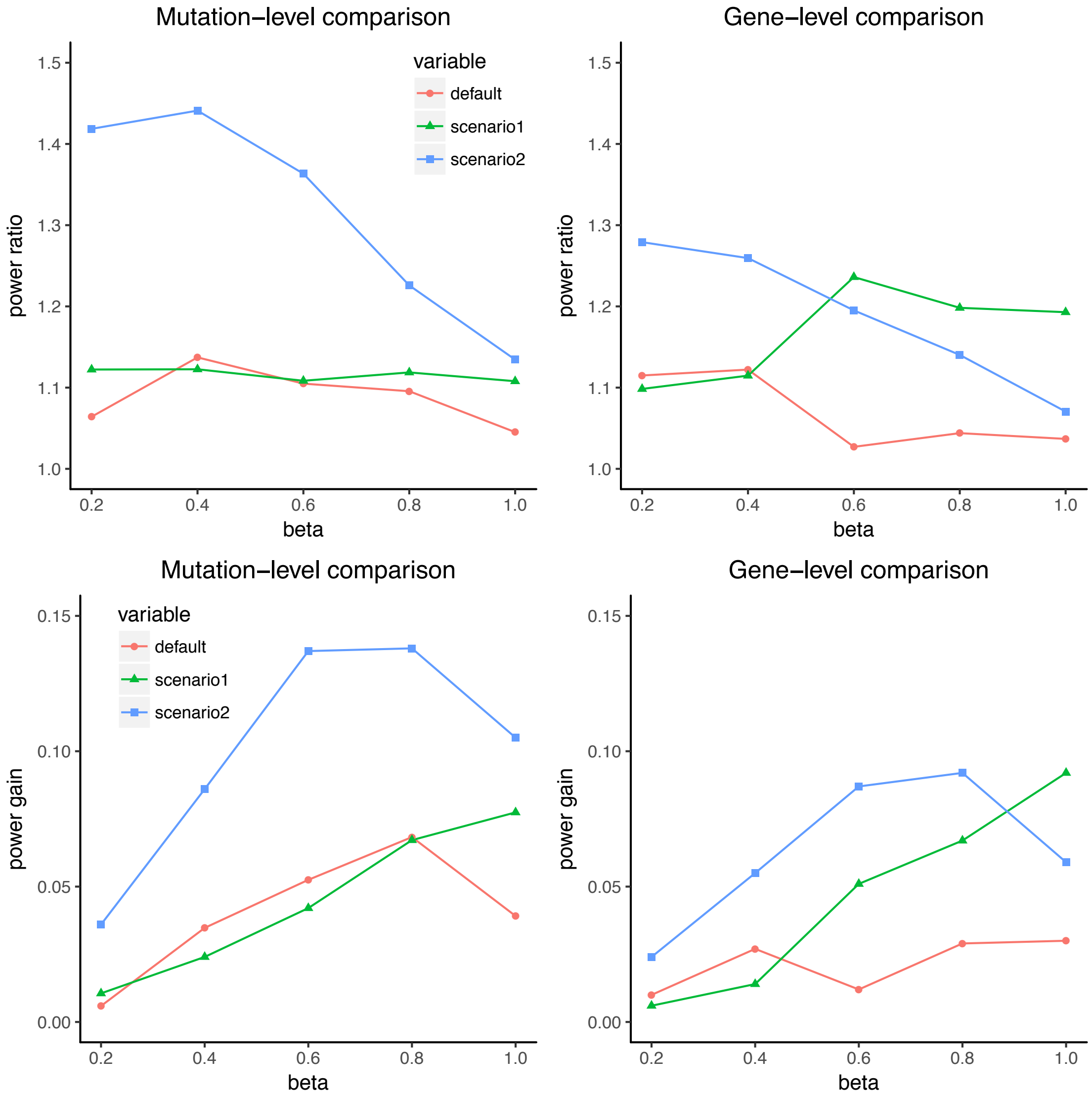

Fig S4: Top: The power ratio (i.e., power of SAME over power of GLM) of the continuous trait with $\rho_1 = 0.05$ for mutation-level comparison and $\rho_1^g = 0.10$ for gene-level comparison. Bottom: The power gain (i.e., the power of SAME minus the power of GLM) of the continuous trait with $\rho_1 = 0.05$ for mutation-level comparison and $\rho_1^g = 0.10$ for gene-level comparison.

Cont. trait	Method	$\beta = 0$	$\beta = 0.2$	$\beta = 0.4$	$\beta = 0.6$	$\beta = 0.8$	$\beta = 1$
$\rho_1 = 0.10$	mSAME	0.050	0.170	0.509	0.825	0.964	0.997
	GLM	0.049	0.162	0.462	0.779	0.941	0.988
$\rho_1 = 0.05$	mSAME	0.049	0.098	0.288	0.553	0.783	0.905
	GLM	0.046	0.092	0.253	0.501	0.715	0.866
$\rho_1 = 0.02$	mSAME	0.053	0.071	0.122	0.200	0.324	0.458
	GLM	0.050	0.070	0.101	0.192	0.297	0.418
Dich. trait	Method	$\beta = 0$	$\beta = 0.4$	$\beta = 0.8$	$\beta = 1.2$	$\beta = 1.6$	$\beta = 2$
$\rho_1 = 0.10$	mSAME	0.047	0.148	0.453	0.784	0.938	0.988
	GLM	0.033	0.140	0.418	0.731	0.895	0.968
$\rho_1 = 0.05$	mSAME	0.056	0.117	0.222	0.457	0.629	0.805
	GLM	0.047	0.102	0.205	0.406	0.578	0.753
$\rho_1 = 0.02$	mSAME	0.052	0.063	0.098	0.152	0.239	0.322
	GLM	0.034	0.047	0.082	0.118	0.203	0.264

Table S1: Power performance of the tests based on 1000 replications with $\alpha = 0.05$ for mutation-level simulations in the main text.

Cont. trait	Method	$\beta = 0$	$\beta = 0.2$	$\beta = 0.4$	$\beta = 0.6$	$\beta = 0.8$	$\beta = 1$
$\rho_1^g = 0.15$	gSAME	0.043	0.148	0.400	0.728	0.921	0.978
	GLM	0.051	0.125	0.386	0.697	0.904	0.974
$\rho_1^g = 0.10$	gSAME	0.051	0.097	0.248	0.455	0.689	0.844
	GLM	0.040	0.087	0.221	0.443	0.660	0.814
$\rho_1^g = 0.05$	gSAME	0.051	0.064	0.105	0.196	0.296	0.426
	GLM	0.057	0.058	0.101	0.154	0.254	0.378
Dich. trait	Method	$\beta = 0$	$\beta = 0.4$	$\beta = 0.8$	$\beta = 1.2$	$\beta = 1.6$	$\beta = 2$
$\rho_1^g = 0.15$	gSAME	0.054	0.112	0.353	0.619	0.820	0.943
	GLM	0.034	0.099	0.335	0.595	0.808	0.928
$\rho_1^g = 0.10$	gSAME	0.042	0.094	0.233	0.424	0.605	0.752
	GLM	0.046	0.094	0.212	0.382	0.572	0.722
$\rho_1^g = 0.05$	gSAME	0.049	0.060	0.093	0.157	0.222	0.313
	GLM	0.047	0.064	0.104	0.156	0.225	0.285

Table S2: Power performance of the tests based on 1000 replications with $\alpha = 0.05$ for gene-level simulations in the main text.

Cont. trait	Method	$\beta = 0$	$\beta = 0.2$	$\beta = 0.4$	$\beta = 0.6$	$\beta = 0.8$	$\beta = 1$
$\rho_1 = 0.10$	mSAME	0.050	0.148	0.428	0.756	0.934	0.989
	GLM	0.059	0.128	0.395	0.699	0.890	0.957
$\rho_1 = 0.05$	mSAME	0.054	0.097	0.220	0.430	0.633	0.795
	GLM	0.050	0.086	0.196	0.388	0.566	0.718
$\rho_1 = 0.02$	mSAME	0.050	0.065	0.089	0.132	0.198	0.260
	GLM	0.049	0.062	0.080	0.114	0.172	0.227
Dich. trait	Method	$\beta = 0$	$\beta = 0.4$	$\beta = 0.8$	$\beta = 1.2$	$\beta = 1.6$	$\beta = 2$
$\rho_1 = 0.10$	mSAME	0.056	0.140	0.377	0.674	0.872	0.970
	GLM	0.049	0.127	0.330	0.619	0.826	0.935
$\rho_1 = 0.05$	mSAME	0.051	0.065	0.163	0.325	0.472	0.626
	GLM	0.047	0.052	0.138	0.268	0.406	0.547
$\rho_1 = 0.02$	mSAME	0.049	0.061	0.086	0.124	0.174	0.226
	GLM	0.044	0.052	0.075	0.109	0.143	0.198

Table S3: Power performance of the tests based on 1000 replications with $\alpha = 0.05$ in mutation-level simulations of scenario (1): with specificity of mutation calls being 0.95 instead of 0.98.

Cont. trait	Method	$\beta = 0$	$\beta = 0.2$	$\beta = 0.4$	$\beta = 0.6$	$\beta = 0.8$	$\beta = 1$
$\rho_1^g = 0.15$	gSAME	0.046	0.093	0.230	0.428	0.638	0.804
	GLM	0.040	0.085	0.201	0.372	0.579	0.737
$\rho_1^g = 0.10$	gSAME	0.051	0.067	0.136	0.267	0.405	0.569
	GLM	0.046	0.061	0.122	0.216	0.338	0.477
$\rho_1^g = 0.05$	gSAME	0.055	0.070	0.086	0.112	0.165	0.246
	GLM	0.054	0.065	0.079	0.106	0.142	0.184
Dich. trait	Method	$\beta = 0$	$\beta = 0.4$	$\beta = 0.8$	$\beta = 1.2$	$\beta = 1.6$	$\beta = 2$
$\rho_1^g = 0.15$	gSAME	0.047	0.088	0.188	0.361	0.535	0.701
	GLM	0.048	0.074	0.170	0.305	0.456	0.610
$\rho_1^g = 0.10$	gSAME	0.052	0.062	0.108	0.202	0.317	0.428
	GLM	0.045	0.054	0.106	0.172	0.255	0.343
$\rho_1^g = 0.05$	gSAME	0.051	0.053	0.071	0.103	0.137	0.161
	GLM	0.065	0.058	0.062	0.080	0.116	0.134

Table S4: Power performance of the tests based on 1000 replications with $\alpha = 0.05$ in gene-level simulations of scenario (1).

Cont. trait	Method	$\beta = 0$	$\beta = 0.2$	$\beta = 0.4$	$\beta = 0.6$	$\beta = 0.8$	$\beta = 1$
$\rho_1 = 0.10$	mSAME	0.046	0.195	0.506	0.837	0.974	0.997
	GLM	0.038	0.145	0.400	0.709	0.930	0.984
$\rho_1 = 0.05$	mSAME	0.057	0.122	0.281	0.514	0.749	0.886
	GLM	0.046	0.086	0.195	0.377	0.611	0.781
$\rho_1 = 0.02$	mSAME	0.051	0.095	0.160	0.233	0.344	0.463
	GLM	0.042	0.073	0.118	0.184	0.264	0.357
Dich. trait	Method	$\beta = 0$	$\beta = 0.4$	$\beta = 0.8$	$\beta = 1.2$	$\beta = 1.6$	$\beta = 2$
$\rho_1 = 0.10$	mSAME	0.051	0.168	0.448	0.765	0.943	0.992
	GLM	0.044	0.125	0.348	0.639	0.857	0.947
$\rho_1 = 0.05$	mSAME	0.061	0.116	0.247	0.446	0.644	0.780
	GLM	0.041	0.090	0.181	0.339	0.506	0.645
$\rho_1 = 0.02$	mSAME	0.060	0.090	0.132	0.198	0.277	0.357
	GLM	0.019	0.040	0.079	0.121	0.165	0.222

Table S5: Power performance of the tests based on 1000 replications with $\alpha = 0.05$ in mutation-level simulations of scenario (2): with lower read-depth to resemble a whole genome sequencing data.

Cont. trait	Method	$\beta = 0$	$\beta = 0.2$	$\beta = 0.4$	$\beta = 0.6$	$\beta = 0.8$	$\beta = 1$
$\rho_1^g = 0.15$	gSAME	0.045	0.137	0.422	0.746	0.925	0.990
	GLM	0.051	0.130	0.355	0.677	0.894	0.971
$\rho_1^g = 0.10$	gSAME	0.047	0.110	0.267	0.533	0.748	0.896
	GLM	0.047	0.086	0.212	0.446	0.656	0.837
$\rho_1^g = 0.05$	gSAME	0.051	0.070	0.137	0.244	0.382	0.535
	GLM	0.047	0.066	0.107	0.175	0.277	0.379
Dich. trait	Method	$\beta = 0$	$\beta = 0.4$	$\beta = 0.8$	$\beta = 1.2$	$\beta = 1.6$	$\beta = 2$
$\rho_1^g = 0.15$	gSAME	0.044	0.132	0.363	0.649	0.876	0.949
	GLM	0.042	0.109	0.303	0.572	0.809	0.921
$\rho_1^g = 0.10$	gSAME	0.050	0.105	0.242	0.466	0.702	0.828
	GLM	0.053	0.092	0.211	0.400	0.590	0.734
$\rho_1^g = 0.05$	gSAME	0.052	0.080	0.127	0.216	0.313	0.436
	GLM	0.057	0.065	0.089	0.157	0.223	0.309

Table S6: Power performance of the tests based on 1000 replications with $\alpha = 0.05$ in gene-level simulations of scenario (2).

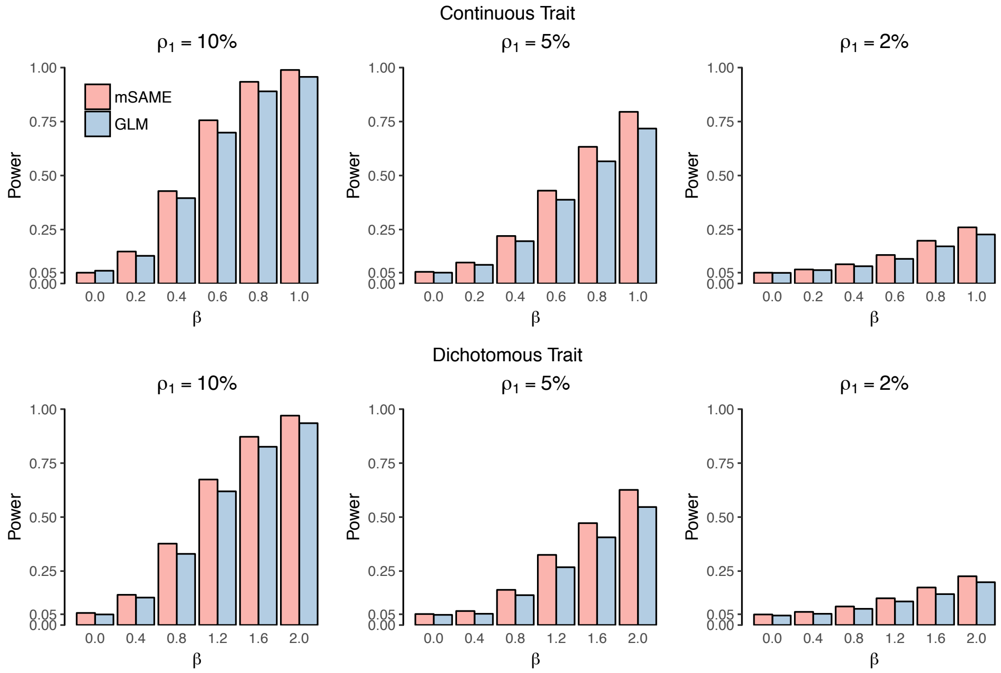

Fig S5: Power comparison of mSAME and GLM for mutation-level simulations in scenario (1).

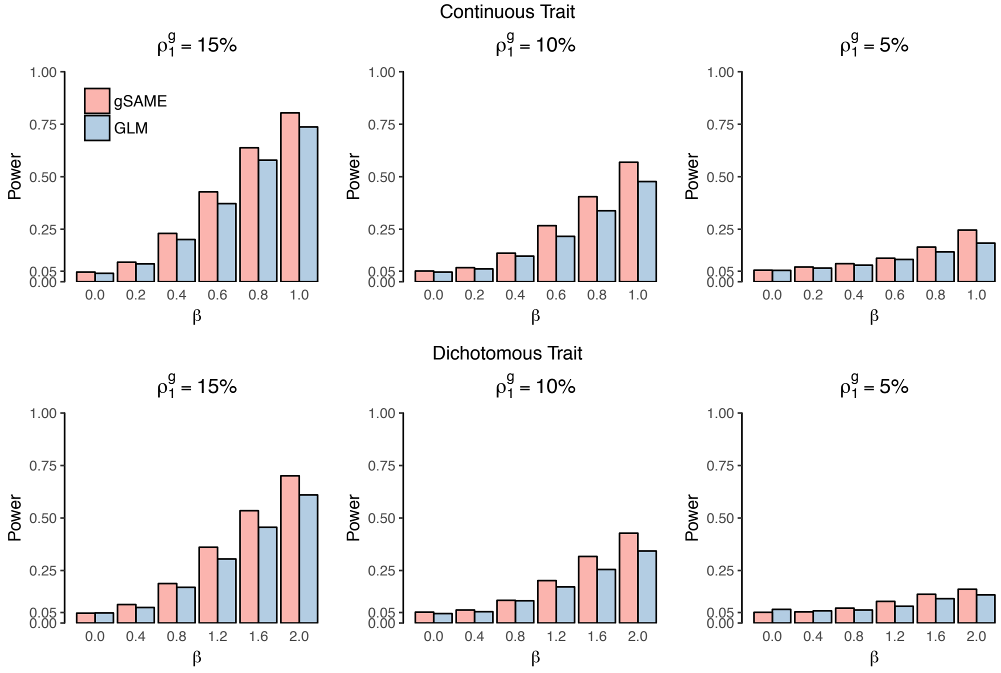

Fig S6: Power comparison of gSAME and GLM for gene-level simulations in scenario (1).

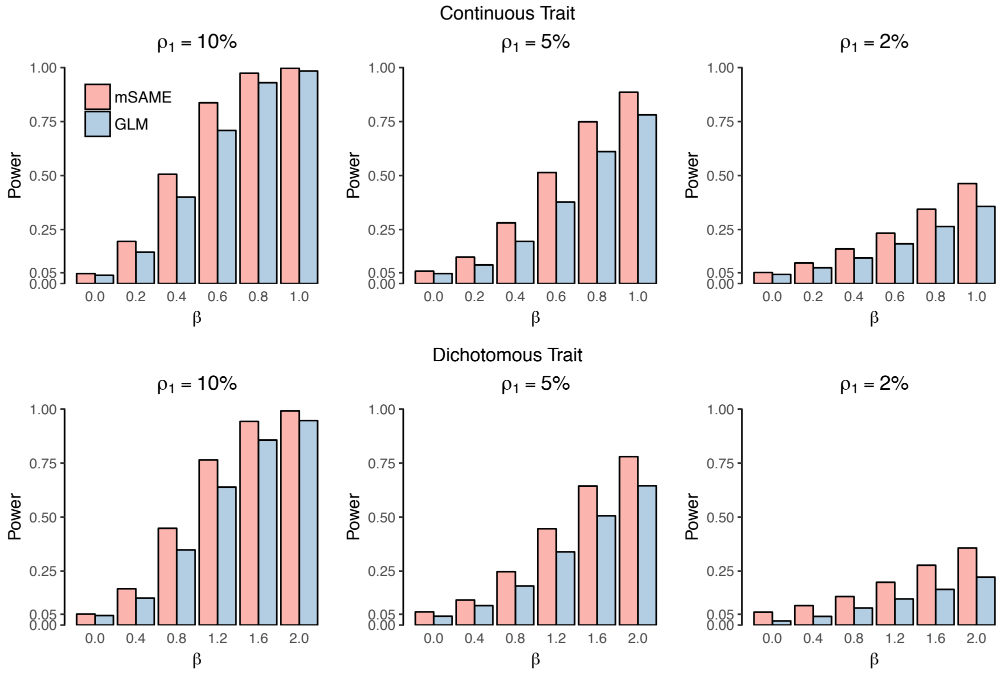

Fig S7: Power comparison of mSAME and GLM for mutation-level simulations in scenario (2).

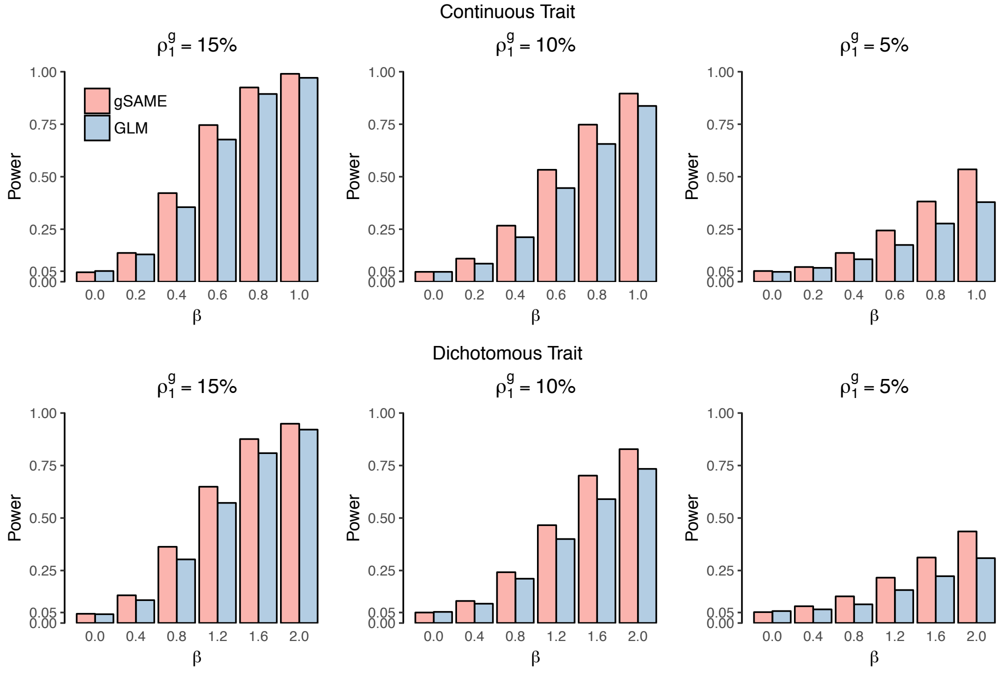

Fig S8: Power comparison of gSAME and GLM for gene-level simulations in scenario (2).
