# Supplementary material for: Association analysis using somatic mutations
Source: PLoS Genet. 2018 Nov 2;14(11):e1007746. doi: 10.1371/journal.pgen.1007746 (PMC6235399; doi:10.1371/journal.pgen.1007746)
Supplement: S3 Appendix — It includes details for the data processing for the real data analysis: (1) Pre-processing and somatic mutation calling; (2) Mutation load and hypermutation status; (3) Allele-specific read counts; (4) Removing copy number effect from gene expression data; (5) Removing potential germline mutations; (6) Mutation frequencies for individual mutations or gene-level mutations; (7) Processing DNA methylation data. (PDF) [file pgen.1007746.s003.pdf]

S3 Appendix. Additional methods/results for real data analysis

3.1 Pre-processing and somatic mutation calling

We first sorted the bam file by read names:

```
samtools sort -n -m 4G --threads 4 -o filename_sort_Qname.bam filename.bam
```

and then preprocess the bam files using an R function `prepareBAM` from R package `asSeq`[1],

```
prepareBAM(input = "filename_sort_Qname.bam",
            outputTag = "filename_sort_Qname_prepBAM",
            filterIt = TRUE, sortIt = FALSE, min.avgQ = 20,
            min.mapQ = 20, getUniqMapping = TRUE)
```

Specifically, we removed all the reads with average sequencing quality score smaller than 20 (`min.avgQ = 20`) or mapping quality smaller than 20 (`min.mapQ = 20`), and also remove non-uniquely mapped reads (`getUniqMapping = TRUE`). Then we sorted the bam file by genomic locations.

We chose to call somatic mutations using both MuTect[2] and Strelka[3] since they have better performance from third party comparison[4]. We called somatic mutations using MuTect (version 1.1.7) with the following command:

```
java -Djava.io.tmpdir=/path_to_tmpdir/ -jar /path2muTect1/muTect-1.1.7.jar \
--disable_auto_index_creation_and_locking_when_reading_rods \
--analysis_type MuTect \
--reference_sequence /path_to_ref/GRCh38.d1.vd1.fa \
--cosmic /path_to_Cosmic/GRCH38_v78/Cosmic_wchr_sorted_by_GRCh38.d1.vd1.vcf \
--dbsnp /path_to_dbSNP/dbsnp_144.hg38_wchr_sorted_by_GRCh38.d1.vd1.vcf \
--input_file:normal /path_to_paired_normal_bam_file \
--input_file:tumor /path_to_paired_tumor_bam_file\
```

```
--out /path_to_output_call_stats_file \
--vcf /path_to_output_vcf_file
```

The reference genome sequence (hg38) were downloaded from GDC website (https://gdc.cancer.gov/about-data/data-harmonization-and-generation/gdc-reference-files). We downloaded Cosmic somatic mutation vcf files for coding and non-coding variants (CosmicCodingMuts.vcf.gz and CosmicNonCodingVariants.vcf.gz) from Cosmic website. Then sort these files according to reference genome and merge them to one vcf file. The dbSNP annotation (version 144) were downloaded from GATK bundle (ftp://gsapubftp-anonymous@ftp.broadinstitute.org/bundle/), and sorted according to the reference genome.

We called somatic mutation using Strelka (version 1.0.14) with the following command:

```
/path_to_strelka_bin/configureStrelkaWorkflow.pl \
--normal=/path_to_paired_normal_bam_file \
--tumor=/path_to_paired_tumor_bam_file \
--ref=/path_to_ref/GRCh38.d1.vd1.fa \
--config=/path_to_strelka_bin/_strelka_config_bwa_default.ini \
--output-dir=/path_to_output_dir

make -C /path_to_output_dir
```

We modified the configuration file by skipping depth filter isSkipDepthFilters=1, according to the recommendation for analyzing exome-seq data using Strelka https://sites.google.com/site/strelkasomaticvariantcaller/home/faq. We took the somatic mutations with a "PASS" status by both MuTec and Strelka, and further filter out the mutations with read-depth lower than 20 in either tumor or paired normal sample or with less than 5 alternative reads in tumor sample.

We further annotated the somatic mutations using ANNOVAR[5], and selected non-silencing mutations, which are are defined as nonsynonymous/stopgain/stoploss exonic mutations or

mutations on splicing sites. We further removed those mutations with Exome Aggregation Consortium (ExAC) minor allele frequency equal to or larger than 10^{-4}. After all these filtering, we ended up with 134,262 mutation calls in 431 samples.

3.2 Mutation load and hypermutation status

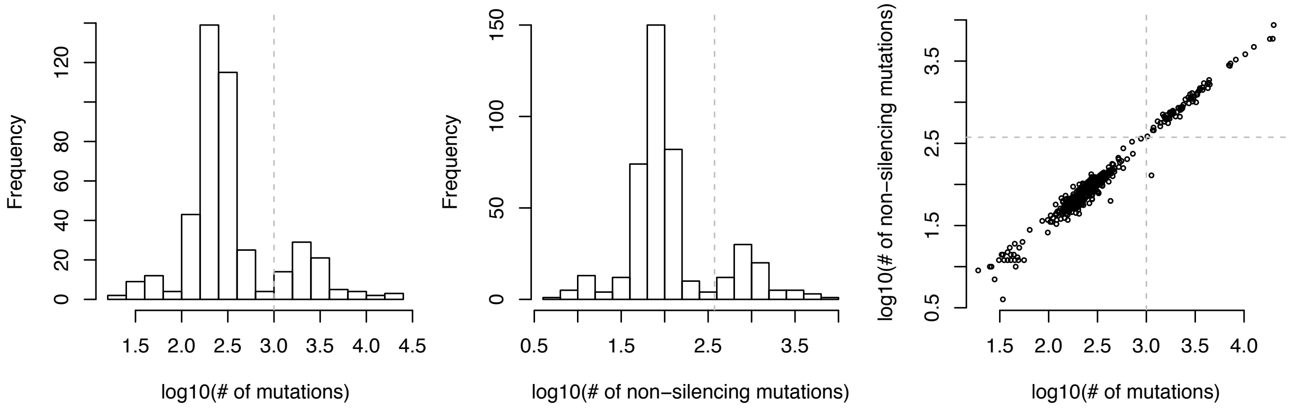

Fig S9: Mutation load across 431 TCGA samples and definition of hyper-mutated samples. Left panel: distribution of the number of mutations per sample across 431 TCGA COAD samples. Middle panel: distribution of the number of non-silencing mutations per sample across 431 TCGA COAD samples. Here non-silencing mutations are defined as those nonsynonymous exonic mutations (including stop gain/loss mutations) and mutations on splicing sites. The right panel shows the scatter plot of these two measurements of mutation load. A sample is claimed as hyper-mutated if there are more than 375 non-silencing mutations (vertical line in the middle panel and horizontal line in the right panel), and this cutoff is very similar to the cutoff of 1,000 total mutations (vertical line in the left panel and the right panel).

3.3 Allele-specific read counts

For each locus, mutation callers only provide allele-specific read counts (ASReCs, i.e., the number of reads that harbor the reference or the alternative/mutant allele) for the samples where the mutation is called. However, our model requires ASReCs for all mutation loci across all samples. We used the `ASEReadCounter` from Genome Analysis Toolkit (GATK) to count allele-specific reads in all the tumor and paired normal samples. We found that 24 samples have low proportion of allele-specific reads in both tumor and normal samples, as shown in

Figure S10. In fact, for these 24 samples, there are no coverage for more than 1/3 of the 134,262 mutation loci (right panel of Figure S10). We removed these 24 samples, and took the intersection of the remaining samples with the samples having gene expression data and obtained 386 samples for the association analysis.

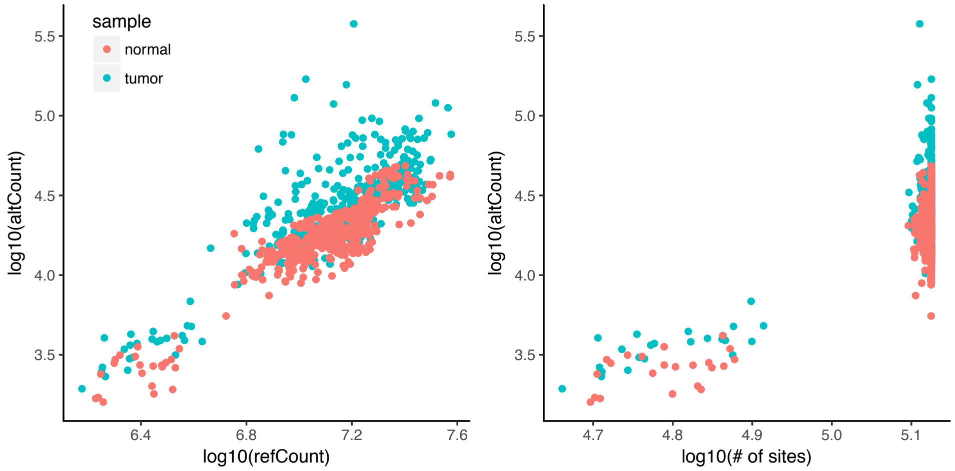

Fig S10: Left panel: The total number of reads with reference allele (x-axis) versus the total number of reads with alternative allele (y-axis) on $\log_{10}$-scale for the tumor and normal samples. Right panel: The total number of mutant loci covered by at least one read versus the total number of reads with alternative allele in $\log_{10}$ scale.

3.4 Removing copy number effect from gene expression data

We downloaded copy number data from NCI GDC data portal using data type "Masked Copy Number Segment" from NCI GDC data portal (`https://portal.gdc.cancer.gov/`). These are text files (`*.nocnv_grch38.seg.txt`), and each file records the segmented copy number measurement for one sample. More specifically, each file includes the genomic regions covered by each segment and the segmental mean value for one tumor sample. These segmental mean values were generated using log2(estimated copy-number/ 2), and thus value 0 indicates no copy number changes and positive/negative values indicates amplification/deletion, respectively. See `https://docs.gdc.cancer.gov/Data/Bioinformatics_Pipelines/CNV_Pipeline/` for details. The word "Maksed" in data type and the word "noncnv" in file names

indicate that known germline copy number variants have been masked. Each segment usually covers a large genomic region of tens or hundreds of genes. We recorded the copy number measurement for each gene as the segmental mean of the segment where this gene is located. Then for each gene, we regressed its gene expression against this copy number measurement across all samples and saved the residuals as copy number corrected measurement of gene expression.

3.5 Removing potential germline mutations

We consider a mutation as a potential germ-line mutation if the numbers of alternative reads in normal samples are generally large. Specifically, we consider a mutation as a potential germline mutation and remove it from our analysis if there are five or more alternative reads at this locus in any normal sample. Figure S11 illustrate an example of somatic mutation (left panel) and an example of germline mutation (right panel). For mutation-level associations, we started with 45 mutations that have occurred in at least 5 of the 386 samples, and then removed 8 potential germline mutations based on this criterion. For gene-level association analysis, we started with 3,444 mutations whose corresponding gene-level mutations are present in at least 5 samples, and then removed 52 potential germline mutations using this criterion.

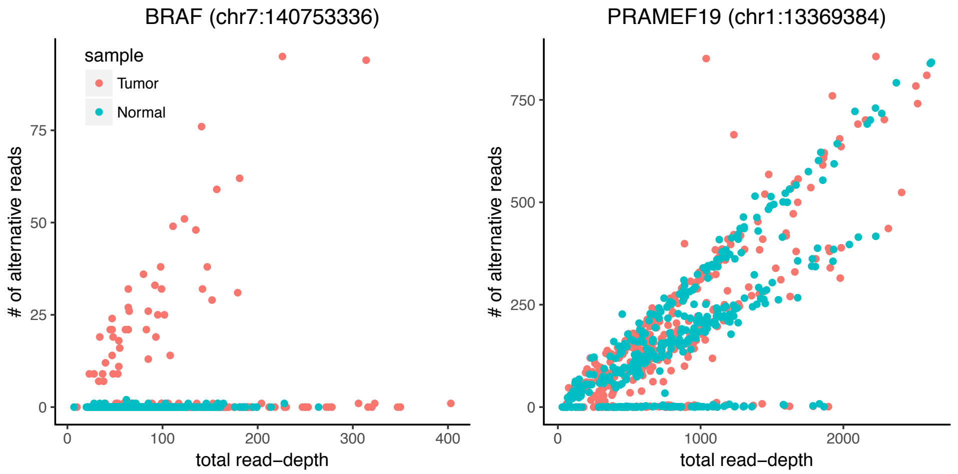

Fig S11: The read depth data of two mutations in both tumor and normal samples. Left panel: A somatic mutation in gene BRAF. The number of alternative reads are smaller than 5 in all the normal samples. Right panel a germline mutation in gene PRAMEF19. The number of alternative reads are equal to or larger than five in 306 normal samples.

3.6 Mutation frequencies for individual mutations or gene-level mutations.

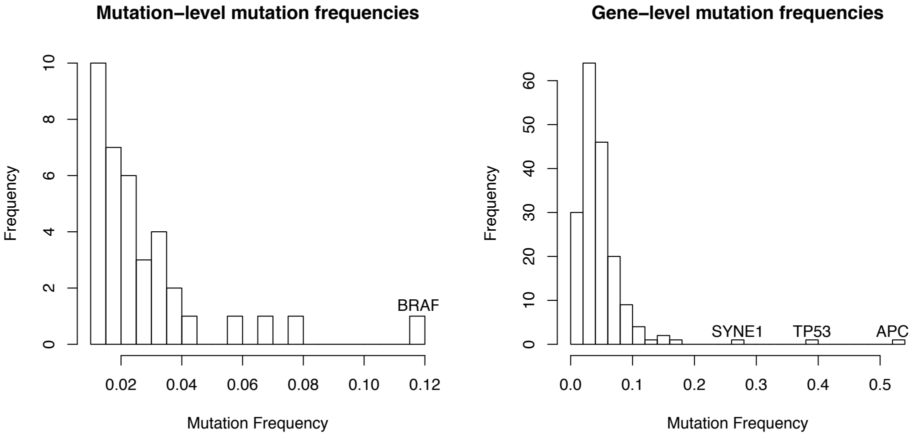

Fig S12: Left panel: The frequencies of all the 37 mutations we used in mutation-level analysis. Right panel: The frequencies of all the 180 gene-level mutations we used in gene-level analysis.

3.7 Supplementary eQTL mapping results in TCGA colon cancer patients.

Table S7: **The summary of the 109 significant results in mSAME model.**

Mutation	Gene	Freq.	Associated genes
chr1:181049121	MR1	1.30	MLH1
chr3:25627236	TOP2B	1.30	C4orf19
chr3:179199088	PIK3CA	3.63	MLH1
chr5:112838007	APC	1.55	ZWILCH
chr7:74824936	GTF2IRD2	2.59	SDR42E1, LINC00675
chr7:140753336	BRAF	11.66	SOX8, AGK, ... (100 genes)
chr17:7673803	TP53	1.55	CDX1
chr17:7674894	TP53	2.33	TP53
chr18:51065549	SMAD4	2.07	TPH1

Table S8: **The summary of the 67 significant results in gene-level association analysis.**

Mutation	Chr	Freq.	Associated genes
ZNF678	1	2.59	MLH1
BIRC6	2	8.03	ENSG00000226320.4
TET3	2	2.59	TOX3, LINC00483
PBRM1	3	4.40	TOX3, IL17RE
FBXW7	4	11.40	TGIF2[2]
ERBB2IP	5	2.59	MLH1
TCF7	5	1.30	C10orf35[2]
BRAF	7	14.25	RPS6KA6, MLH1, EPDR1, LAPTM4B, RNF43, KLF7, RAB32, MTERF1, KHDRBS3, VAV3, EMX1, TTPA, GGH, PROSER2, CDHR1, PTPRD, CDX2, AXIN2, CELP, ENSG00000175509.8, TRAK1, TRMT12, RNLS, RBM11, DNAH14, NHLRC1, C10orf99, GSPT2, TBC1D8, PTPRD-AS1, SATB2-AS1, ENSG00000227359.1, GUSBP5, ENSG00000236935.1, TRNP1[1], LINC00668
PTEN	10	4.15	IFNLR1
POLE	12	5.96	MLH1
LMO7	13	5.96	MLH1
DYNC1H1	14	7.77	HNF1B[2]
MAP2K1	15	1.81	ASB14
AXIN1	16	1.81	PRSS3
TP53	17	39.38	FAS[1], DDB2, MDM2, SPATA18, LY6D, ZMAT3[1], TSPYL1[2], ENSG00000234546.2, PTCHD4, LINC01021, ENSG00000251095.5,
TGIF1	18	2.07	TGIF1
ZNF521	18	5.96	MLH1
SMAD2	18	4.15	HOXA10
ZNF99	19	5.18	MLH1[1]
KDM6A	X	2.07	GLOD5

The associated genes identified by gSAME only is labeled with subscript 1, and the associated genes identified by GLM only is labeled with subscript 2. Ensembl gene ids are used when the official gene symbols do not exist.

3.8 DNA methylation data processing and analysis

We downloaded DNA methylation data using data type "Methylation Beta Value". These are text files recording the so called beta-value, which takes values from 0 to 1, with 0 means no methylation and 1 means maximum methylation in the tissue sample. Note that methylation status of a specific CpG within a cell is a binary event. However, when methylation is measured in a tissue sample with millions of cells, the beta value of a CpG reflects the proportion of cells where this CpG is methylated. Two platforms are used to measure methylation data in colon cancer patients: Illumina 27k array and 450k array, which measured the DNA methylation for around 27,000 and 450,000 CpG's, respectively. We will only consider the CpGs that are measured by both platforms. There could be systematic difference between the measurements of these two platforms. We correct such batch effect in the following steps. First, we convert the beta-value to m-value by a logistic transformation to avoid the constrained range of beta-values. Next, for each CpG, we calculated the median value of DNA methylation for each CpG in the two platforms. Then we fit a loess curve of the medians of all the CpGs for the two platforms, and use this loess fit to correct the difference between the two platforms. Finally, we converted the adjusted m-values back to beta-values.

We further removed all the probes with more than 10% of missing values in 27k or 405k platform and ended up with 23,309 probes across 448 samples. To reduce variations of the clustering results, we removed the methylations which are on chromosome X or Y, or have standard deviations less than 0.2 across these samples, and kept the remaining 2,059 methylation probes. Next we applied hierarchical cluster with average linkage, and divided these 448 samples into 2 clusters. We conducted our association analysis using 385 samples with both methylation and somatic mutation data.

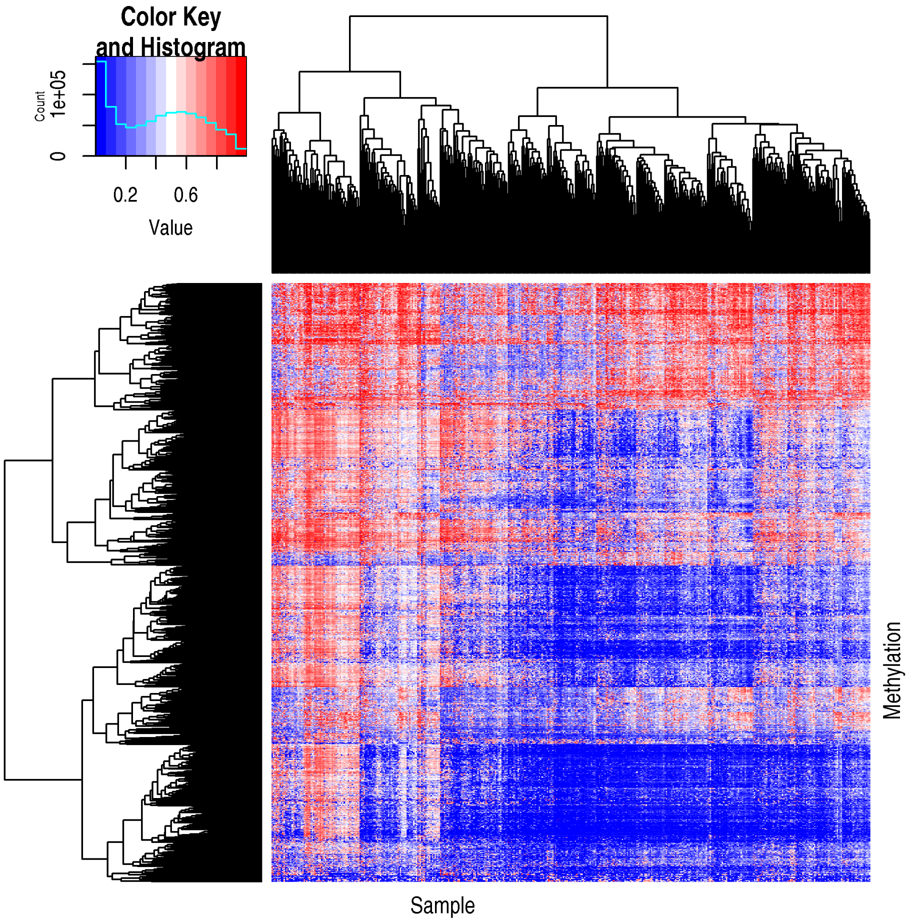

Fig S13: Heatmap of the methylation data.

3.9 Details for using the cloud platform to obtain the read-count data

For those 11 additional TCGA cancer types we analyzed in section "eQTL Analysis for Pan-Cancer Studies", we obtained the read-count data directly from the cloud platform provided by The Seven Bridges Cancer Genomics Cloud. Specifically, on the cloud platform, we added the BAM files of exome-seq data for each cancer type to a project, and uploaded a VCF file including the mutation sites we obtained from the somatic mutation data. Then we applied the ASEReadCounter of GATK on the cloud to count the allele-specific reads in tumor and normal samples for these cancer types. The cloud platform employs the Amazon Web Services (AWS) for the computation and storage of the data. It costs around $0.20 and 40 minutes for each counting job.

3.10 Supplementary eQTL mapping results in TCGA pan-cancer study

Table S9: **The summary of the number of the significant results for 12 TCGA cancer types.**

		Mutation-level				Gene-level			
Cancer	Ng	Nm	GLM	mSAME	com	Nm	GLM	gSAME	com
BLCA	19358	9	71	235	71	206	287	1093	281
COAD	16339	37	100	109	100	180	63	63	59
GBM	20480	1	172	190	172	90	7	32	2
HNSC	18916	18	0	29	0	84	297	302	289
KIRC	20398	0	0	0	0	6	15	14	13
LGG	20524	9	1	173	1	7	27	18	12
LIHC	17784	3	3	116	3	31	240	338	229
LUAD	20297	4	38	65	38	490	540	253	228
LUSC	20637	7	1	2	1	393	67	43	43
OV	22079	1	0	0	0	37	3	1	1
SKCM	19169	264	17	60	16	1641	35	56	27
STAD	22800	10	9	16	9	393	830	788	662

For each cancer type, Ng represents the number of gene expression traits, Nm represents the number of mutations used for mutation-level or gene-level association analysis, and com represents the number of association results shared between GLM and SAME.

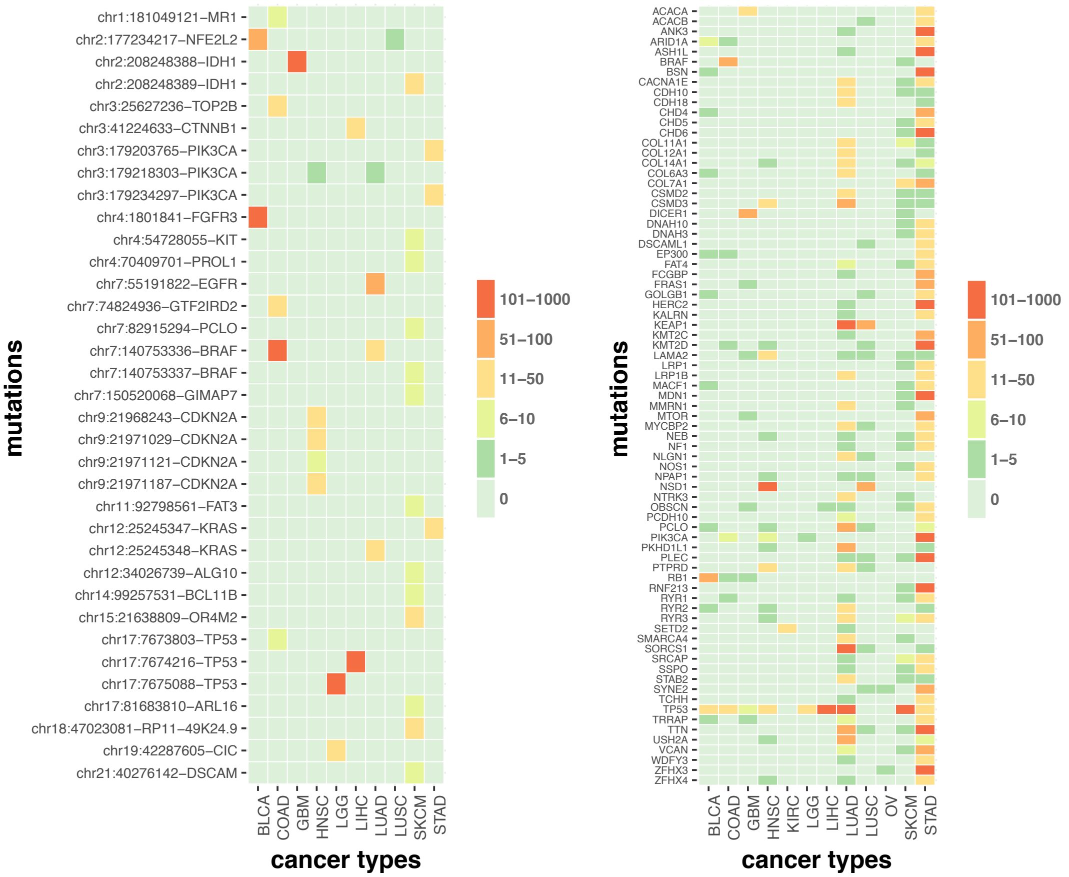

Fig S14: Summary of pan-cancer eQTL mapping results by SAME using transcriptome-wide correction (i.e., p-value cutoff 0.05/# of gene expression traits). Left panel: a heatmap of mutation-level eQTL mapping results by mSAME across cancer types. Each cell in the heatmap is colored according to the number of significant associations for one mutation (row) in one cancer type (column). Only those mutations that are associated in 6 or more genes across the 12 cancer types are shown. Right panel: a heatmap of gene-level eQTL mapping results by gSAME across cancer types. Only those gene-level mutations that have eQTL in more than one cancer type, and are associated with 21 or more genes across the 12 cancer types are shown.

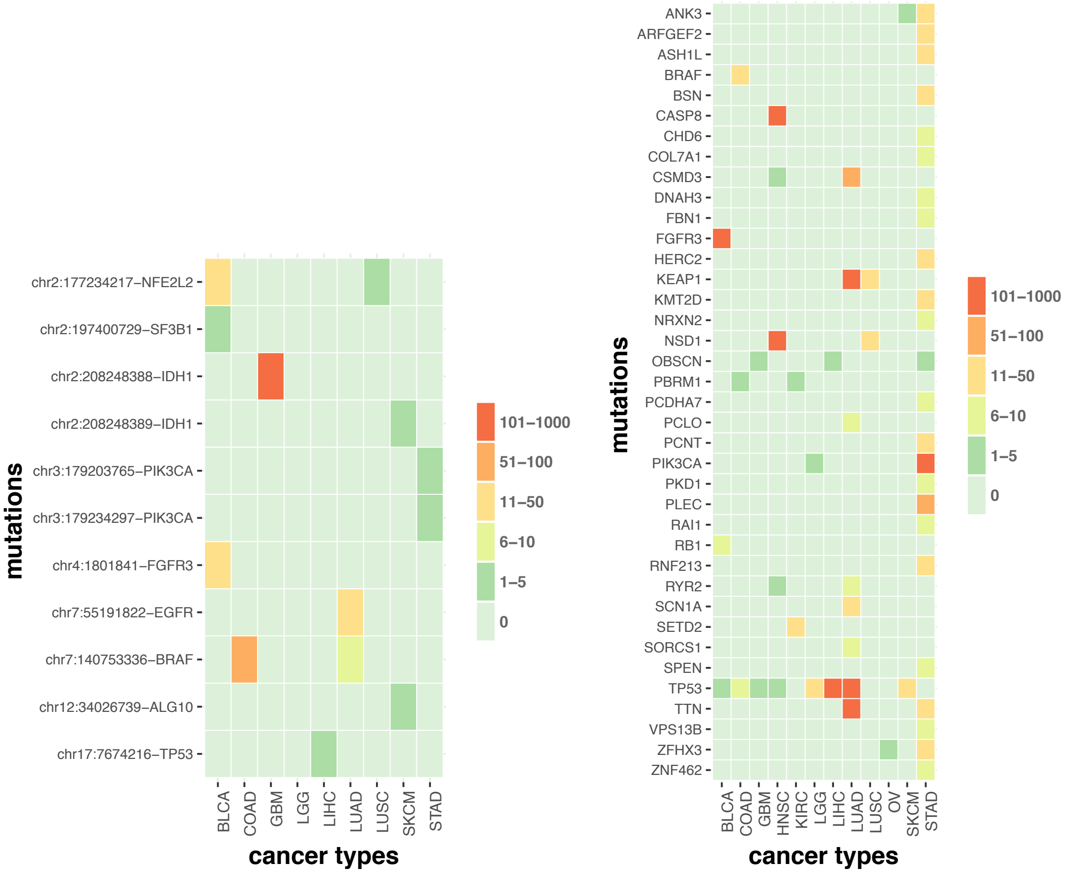

Fig S15: Summary of pan-cancer eQTL mapping results by GLM using Bonferroni correction. In left panel, mutations that are associated with 2 or more genes across the 12 cancer types are shown. In right panel, gene-level mutations that are associated with 6 or more genes across the 12 cancer types are shown.

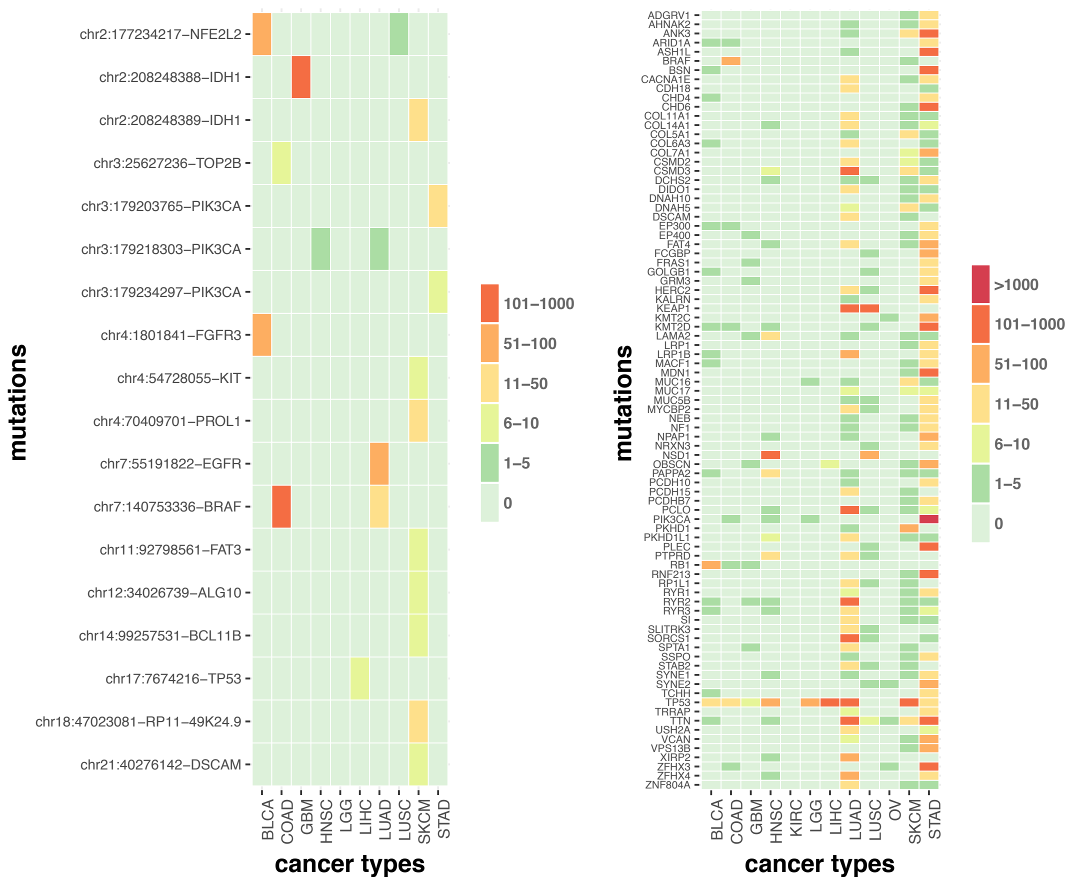

Fig S16: Summary of pan-cancer eQTL mapping results by GLM using transcriptome-wide correction. In left panel, only those mutations that are associated in 6 or more genes across the 12 cancer types are shown. In right panel, only those gene-level mutations that have eQTL in more than one cancer type, and are associated with 21 or more genes across the 12 cancer types are shown.

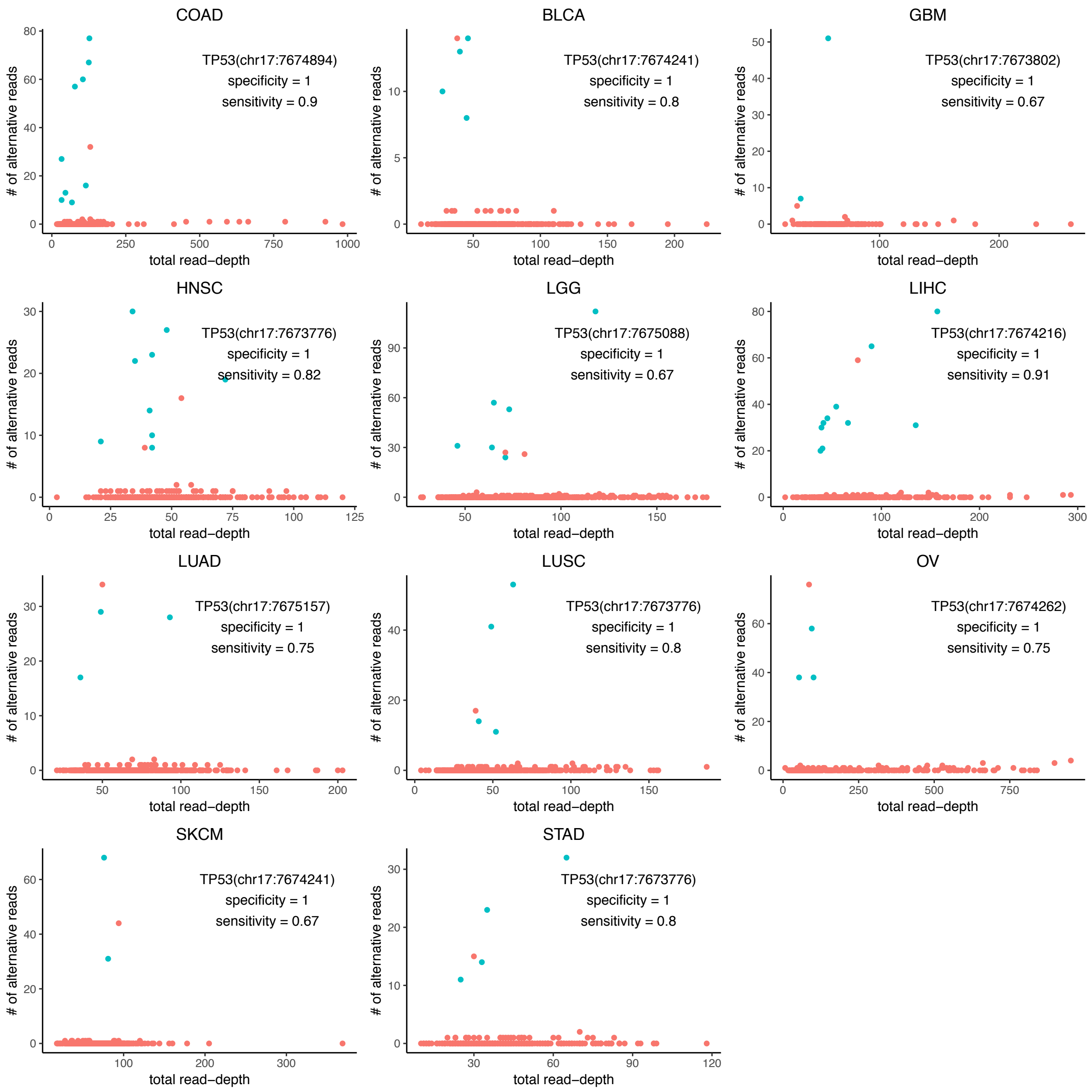

Fig S17: Examples of potential somatic mutation calling errors for mutations of TP53. Each point indicates the read-depth (x-axis) and the number of alternative reads (y-axis) in one sample. Blue and red colors indicate with or without somatic mutation call.

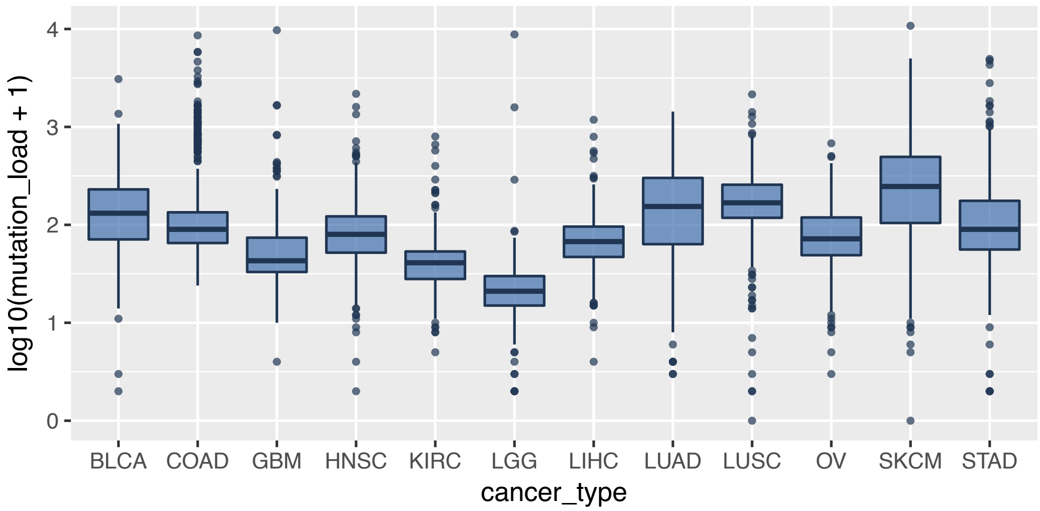

Fig S18: Mutation load (total number of somatic mutations per sample) across cancer types.

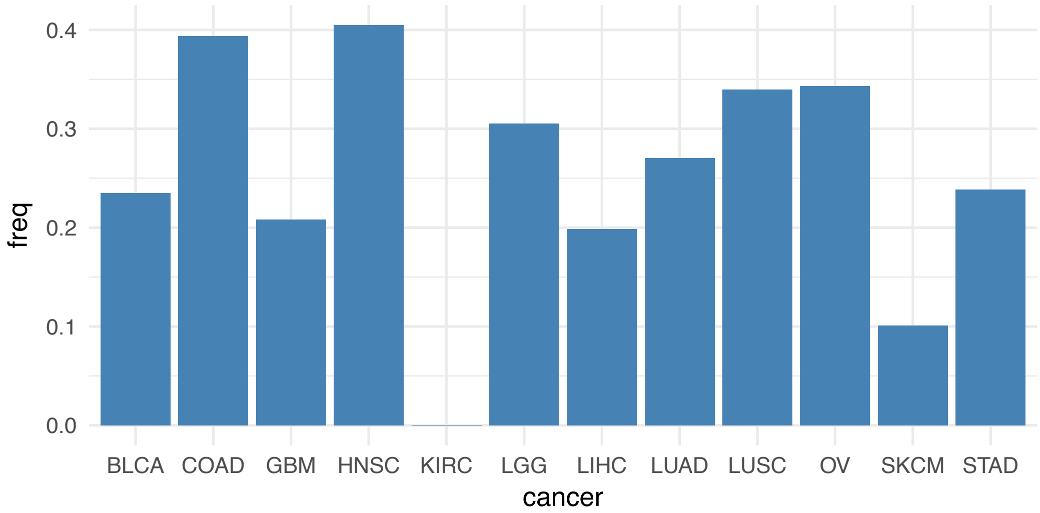

Fig S19: Mutation frequency of TP53 across cancer types.

References

1. Sun W. A statistical framework for eQTL mapping using RNA-seq data. Biometrics. 2012;68(1):1–11.

2. Cibulskis K, Lawrence MS, Carter SL, Sivachenko A, Jaffe D, Sougnez C, et al. Sensitive detection of somatic point mutations in impure and heterogeneous cancer samples. Nature Biotechnology. 2013;31(3):213–219.

3. Saunders CT, Wong WS, Swamy S, Becq J, Murray LJ, Cheetham RK. Strelka: accurate somatic small-variant calling from sequenced tumor–normal sample pairs. Bioinformatics. 2012;28(14):1811–1817.

4. Xu H, DiCarlo J, Satya RV, Peng Q, Wang Y. Comparison of somatic mutation calling methods in amplicon and whole exome sequence data. BMC Genomics. 2014;15(1):244.

5. Wang K, Li M, Hakonarson H. ANNOVAR: functional annotation of genetic variants from high-throughput sequencing data. Nucleic Acids Research. 2010;38(16):e164–e164.
